# High-throughput quantification of quasistatic, dynamic and spall strength of materials across 10 orders of strain rates

**DOI:** 10.1093/pnasnexus/pgae148

**Published:** 2024-04-05

**Authors:** Suhas Eswarappa Prameela, Christopher C Walker, Christopher S DiMarco, Debjoy D Mallick, Xingsheng Sun, Stephanie Hernandez, Taisuke Sasaki, Justin W Wilkerson, K T Ramesh, George M Pharr, Timothy P Weihs

**Affiliations:** Department of Materials Science and Engineering, MIT, Cambridge, MA 02139, USA; Department of Aeronautics and Astronautics, MIT, Cambridge, MA 02139, USA; Department of Materials Science and Engineering, Johns Hopkins University, Baltimore, MD 21218, USA; Hopkins Extreme Materials Institute, Johns Hopkins University, Baltimore, MD 21218, USA; Department of Materials Science and Engineering, Texas A&M University, College Station, TX 77843, USA; Hopkins Extreme Materials Institute, Johns Hopkins University, Baltimore, MD 21218, USA; Sindri Materials Corp., West Chester, PA 19382, USA; Hopkins Extreme Materials Institute, Johns Hopkins University, Baltimore, MD 21218, USA; DEVCOM Army Research Laboratory, Aberdeen Proving Ground, MD, 21005-5066, USA; Department of Mechanical and Aerospace Engineering, University of Kentucky, Lexington, KY 40506, USA; Department of Mechanical Engineering, Johns Hopkins University, Baltimore, MD 21218, USA; National Institute for Materials Science, Tsukuba 305-0047, Japan; Center for Elements Strategy Initiative for Structural Materials, Kyoto University, Kyoto 606-8501, Japan; Department of Materials Science and Engineering, Texas A&M University, College Station, TX 77843, USA; J. Mike Walker ’66 Department of Mechanical Engineering, Texas A&M University, College Station, TX 77843, USA; Hopkins Extreme Materials Institute, Johns Hopkins University, Baltimore, MD 21218, USA; Department of Mechanical Engineering, Johns Hopkins University, Baltimore, MD 21218, USA; Department of Materials Science and Engineering, Texas A&M University, College Station, TX 77843, USA; Department of Materials Science and Engineering, Johns Hopkins University, Baltimore, MD 21218, USA; Hopkins Extreme Materials Institute, Johns Hopkins University, Baltimore, MD 21218, USA

**Keywords:** high-throughput, strain rate, dynamic behavior, spall, microstructure design

## Abstract

The response of metals and their microstructures under extreme dynamic conditions can be markedly different from that under quasistatic conditions. Traditionally, high strain rates and shock stresses are achieved using cumbersome and expensive methods such as the Kolsky bar or large spall experiments. These methods are low throughput and do not facilitate high-fidelity microstructure–property linkages. In this work, we combine two powerful small-scale testing methods, custom nanoindentation, and laser-driven microflyer (LDMF) shock, to measure the dynamic and spall strength of metals. The nanoindentation system is configured to test samples from quasistatic to dynamic strain-rate regimes. The LDMF shock system can test samples through impact loading, triggering spall failure. The model material used for testing is magnesium alloys, which are lightweight, possess high-specific strengths, and have historically been challenging to design and strengthen due to their mechanical anisotropy. We adopt two distinct microstructures, solutionized (no precipitates) and peak-aged (with precipitates) to demonstrate interesting upticks in strain-rate sensitivity and evolution of dynamic strength. At high shock-loading rates, we unravel an interesting paradigm where the spall strength vs. strain rate of these materials converges, but the failure mechanisms are markedly different. Peak aging, considered to be a standard method to strengthen metallic alloys, causes catastrophic failure, faring much worse than solutionized alloys. Our high-throughput testing framework not only quantifies strength but also teases out unexplored failure mechanisms at extreme strain rates, providing valuable insights for the rapid design and improvement of materials for extreme environments.

**Significance Statement**

Metals are integral to modern life, but their performance in extreme dynamic environments remains a critical concern. Unfortunately, traditional techniques for testing these materials, such as Kolsky bars and large spall experiments, are expensive, low throughput, and highly destructive to both the material and testing setup. Here, we present a high-throughput testing framework to quantify the mechanical strength of metals across 10 orders of strain rate. By leveraging custom nanoindentation and laser-driven microflyer impact system, we show the dynamic strength of peak-aged magnesium alloys converges to that of solutionized counterparts as the strain rate increases, ultimately failing catastrophically due to precipitates under spall.

## Introduction

Traditionally, the mechanical properties of bulk structural materials across strain-rate regimes have been probed using bulk techniques. For the quasistatic strain-rate regime, this evaluation has been done through tension, compression, bending, and torsion experiments. For the dynamic strain rate regime, researchers have employed plate impact, shock, isentropic compression, and Kolsky bar experiments (1). These test protocols are useful for bulk samples but pose challenges when rapid screening of properties is needed for materials design and discovery for extreme environments (2). Furthermore, some large-scale shock/high strain rate experiments can be highly destructive, expensive, and logistically burdensome to implement. There have been several efforts to implement small-scale mechanical testing methods, both at the microscale and nanoscale. Microtensile, microcantilever, and micropillar tests and their counterparts at the nanoscale have allowed researchers to carry out site-specific or volume-specific experiments and obtain the attendant mechanical response (3–5). These experiments have also been coupled with various diagnostic tools such as scanning electron microscopy (SEM), transmission electron microscopy (TEM), and other X-ray or beamline instruments that offer insights into deformation mechanisms and in situ tracking of key parameters such as texture/precipitate evolution, extent of slip behavior, and twin volume fractions (6–8) in metals.

Over the last two decades, several efforts have pushed small-scale mechanical testing protocols to adopt testing conditions to recapitulate those encountered in extreme environments. For example, there are several active efforts to push the popular nanoindentation technique to high strain-rate regimes often encountered during car crashes and ballistic impact. These efforts have focused on improving the calibration methods, measurement strategies, noise reduction techniques, and expanding the range of material systems that can be tested (9–13). Some studies have looked at the impact of continuous stiffness measurement (CSM) methods on hardness overestimation and subsequent challenges in measuring strain-rate sensitivity (14). Managing noise levels and data analysis become especially challenging at high strain rates. Advanced sensors and high-frequency modulation techniques are being developed to circumvent these challenges.

There have also been several efforts to mimic shock-loading conditions through launched particles and plates. These impact experiments cause materials to experience deformation at high strain rates. For example, laser-induced particle impact test (LIPIT) experiments have been successfully implemented to test various metallic alloys, ceramics, and other structural materials (15, 16). In these experiments, a laser is used to accelerate microparticles (∼10 to 50 μm) at varying speeds (∼100 to 900 ms^−1^) toward a target material. These experiments have helped to elucidate adhesion mechanisms, cold spray mechanisms, recrystallization, plasticity, and damage at extreme strain rates. Similarly, laser energy can be used to accelerate thin circular metal disks to mimic plate impact and interrogate spall behavior. Several recent studies have looked at laser-driven microflyer shock experiments for testing various metallic alloys, single crystals, ceramic carbides, and other structural materials (17–19).

**Fig. 1.**
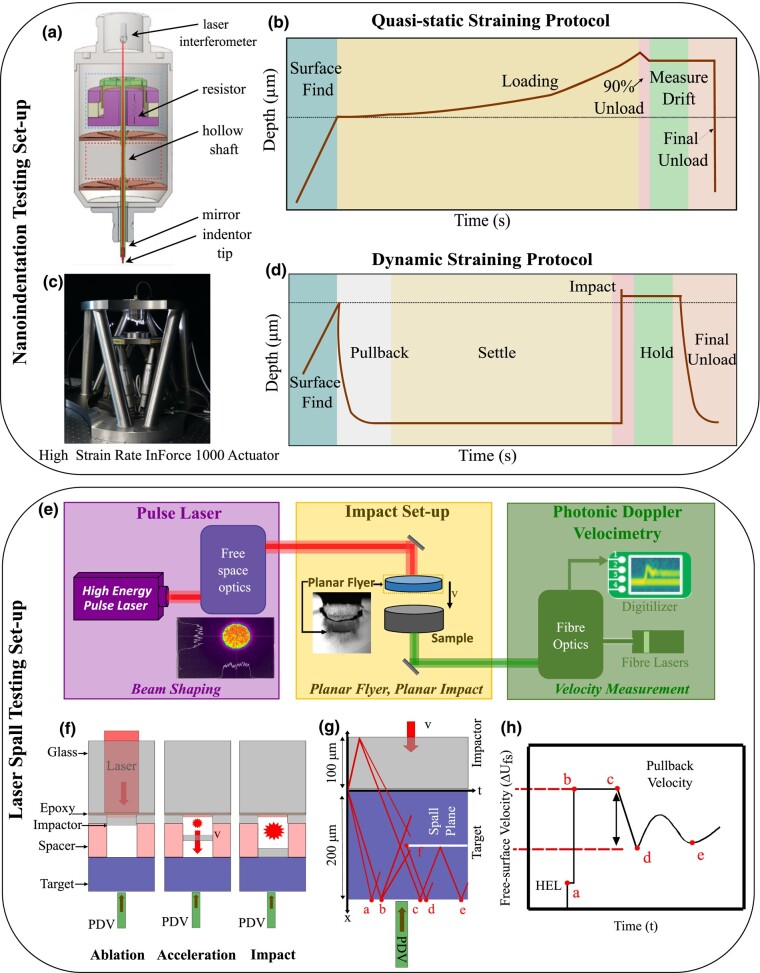
Schematic of nanoindentation setup a) and c) with typical depth vs. time plots for b) quasistatic and d) dynamic loading protocols. e) Schematic of LDMF shock setup with expected results collected to calculate spall strength. The overall layout of the testing system is broken into three major sections: the pulse laser, the impact setup, and the photonic Doppler velocimetry system. f) A side view schematic of the layered impact setup depicting a typical single impactor-target configuration. This shows the three stages of the launch process: ablation of the epoxy, acceleration of the flyer, and finally, impact with the target. g) A view of a position vs. time Lagrangian diagram depicting the propagation of waves throughout the impactor and target that leads to the spall event, as well as the critical points measured via Photonic Doppler Velocimetry (PDV). h) An idealized velocity vs. time plot calculated from the PDV measurements on the target’s free surface. The critical points correspond to the ones shown in g.

In this study, we chose a magnesium (Mg) alloy as the model material to demonstrate rapid quantification of strength across various strain rates and link them to attendant microstructures and plasticity mechanisms. Our testing framework (Fig. 1) employs custom nanoindentation and laser-driven microflyer (LDMF) shock experiments to help quantify the quasistatic, dynamic, and spall strength of these metallic alloys. To explore the effect of heterogeneous inclusions, such as precipitates, we apply similar testing protocols on two different variants of the same metallic alloy, peak-aged samples (with precipitates), and solutionized samples (without precipitates). High-throughput experiments can accelerate the testing of these variants while shedding insights into how microstructural features such as precipitates that are conventionally found to be favorable for metal strengthening at quasistatic strain rates behave in dynamic and spall regimes.

When Mg alloys are deformed at high strain rates ($∼10^{4-8}$ s^−1^) through impact experiments, shockwaves are generated that first load the material in uniaxial compression. When these shock waves meet free surfaces, they reflect as rarefaction fans that can intersect within the material, resulting in dynamic tensile loading that is nearly hydrostatic. This dynamic hydrostatic tension causes the material to fail through a process called spallation, with such spall failures typically driven in metals by void nucleation, growth, and coalescence. The high specific strength of Mg alloys offers a compelling reason to pursue directions related to enhancing spall strength (20). The spall strength, defined as resistance to spallation, strongly depends on microstructural features such as grain size, texture, precipitate type and volume fraction. The spall strength is also a strong function of the tensile strain rate and potentially of the degree of shock compression before the tensile loading occurs. While there have been studies on the dynamic and spall failure of Mg single crystals and alloys (19, 21–25), there is still a lack of clarity on the microstructure property linkages. Recent spall studies in Mg alloys have shown the importance of precipitate size and distribution. In the case of AZ31 (∼Mg–3Al–1Zn) alloy, some large-size precipitates result in catalytic void nucleation and accelerated spall failure in the materials (24, 26). Taken together, these prior studies make a strong case to employ high-throughput techniques to quickly quantify spall strength and to use that data along with the quasistatic and dynamic strengths of Mg alloys to understand the microstructure–property linkages in these materials.

## Results and discussion

### Initial microstructure characterization results

**Fig. 2.**
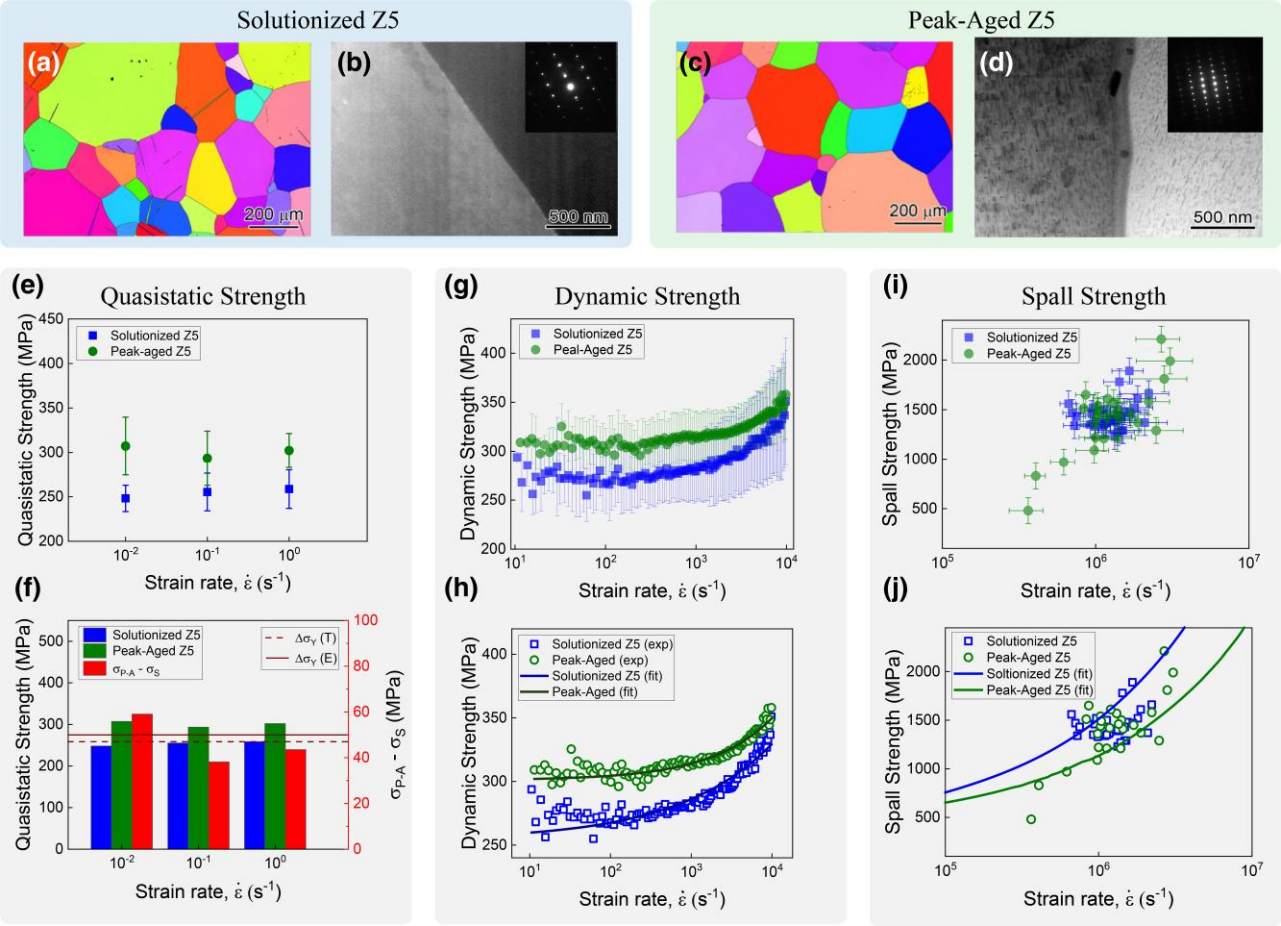
a) EBSD map of Z5 solutionized alloy at $150^{\circ }$C. b) STEM images of Z5 solutionized alloy at $150^{\circ }$C. c) EBSD map of Z5 peak-aged at $150^{\circ }$C. d) STEM images of Z5 peak-aged at $150^{\circ }$C. e) Quasistatic strength measured via nanoindentation with error bars. f) Theoretical and experimental values of strength at quasistatic strain rates. g) Dynamic strength measured via nanoindentation. h) Theoretical dynamic strength predictions using the Zerilli–Armstrong model. i) Spall strength measured via laser spall with uncertainty of spall strength parallel to the *y*-axis and uncertainty of strain rate parallel to the *x*-axis. j) Spall strength predictions using the Wilkerson–Ramesh model

An Mg–5Zn (at%) alloy, referred to as Z5 hereafter, was processed in two conditions: solutionized and peak-aged. Electron backscattered diffraction (EBSD) of these two samples (Fig. 2a and c) showed the average grain size was around ∼205 μm for the Z5 solutionized (without precipitates) and ∼227 μm for the Z5 peak-aged sample (with precipitates). Furthermore, scanning transmission electron microscope (STEM) micrographs (Fig. 2b and d) showed that Z5 solutionized was devoid of any precipitates within and along grain boundaries while the Z5 peak-aged sample had uniformly distributed precipitates from the aging treatment. The precipitate length is around ∼52 nm, and the areal density is ∼390 precipitates per $\mu {\text{m}}^{2}$. Additional STEM TEM studies were conducted to characterize the size and distribution of precipitate microstructures, and additional TEM micrographs are shown in Fig. S7a and b.

### Nanoindentation results—mechanical properties and microscopy

**Fig. 3.**
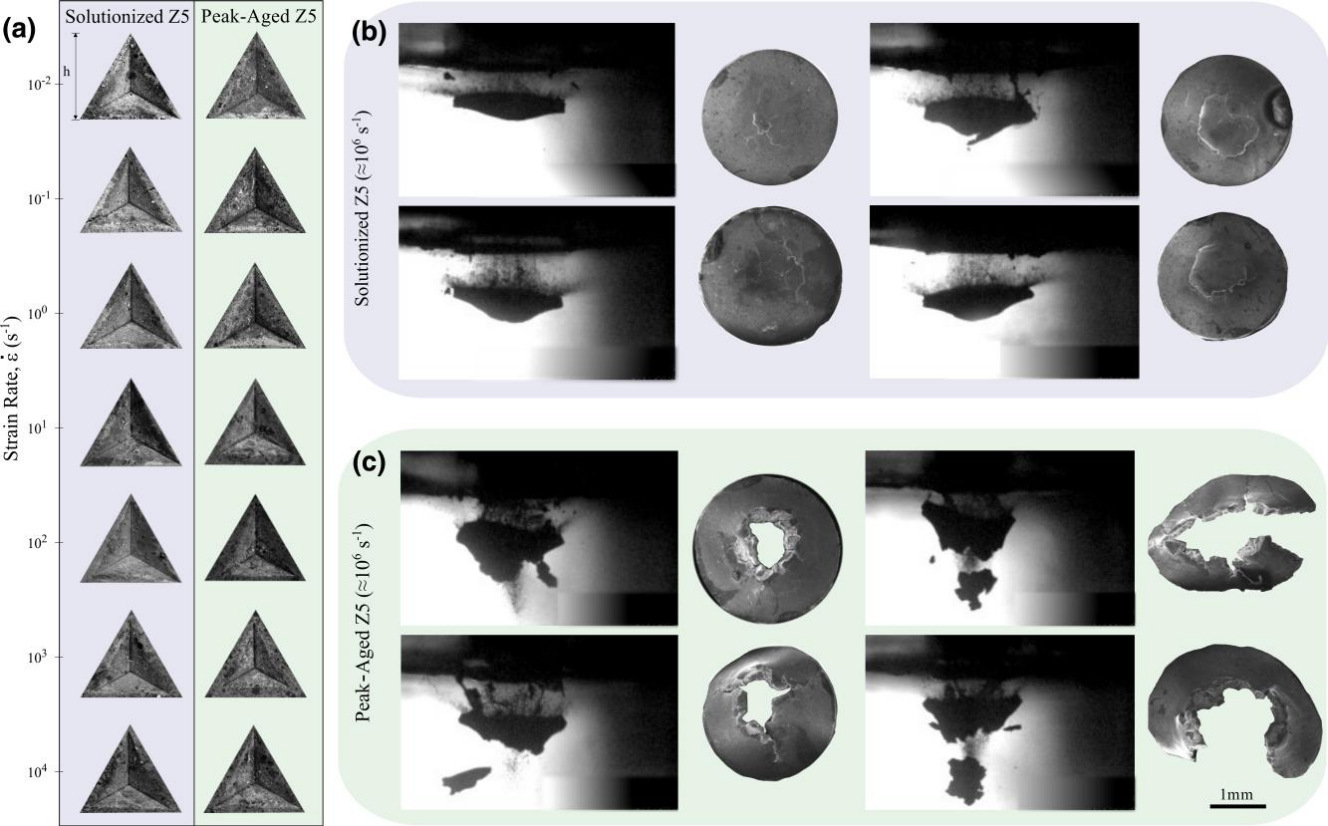
a) Nanoindentations of peak-aged and solutionized Z5 samples at quasistatic and dynamic strain rates. h=21, 21, 21, 59, 59, 29, 39μm for each indentation at each strain rate, from low to high strain rates, respectively. High-speed video shots and postmortem SEM samples of b) Z5 solutionized, c) Z5 peak-aged after undergoing spall. The scale bar of 1 mm applies to all images shown in b and c.

A custom nanoindentation protocol (Fig. 1a–d) was used to probe the mechanical properties at quasistatic and dynamic strain-rate regimes. The indents across the strain-rate regimes varied in size and are shown in Fig. 3a as a function of discrete strain rates. The nanoindentation hardness was measured between strain rates of ($10^{-3}$ to $10^{+4}\;s^{-1}$) as shown in Fig. S3e and f. The hardness values were then converted to strength by a conversion factor of 1/3 (27, 28), and a graph of nanoindentation strength as a function of strain rate was plotted in (Fig. 2e and g). This conversion factor was proposed by Tabor based on analyzing the slip line field of indentation to link yield stress to hardness values (29). It has been used in several studies related to ideal plastic materials and several common metals and their alloys, which possess significant work hardening capacity (30). Studies report challenges in fitting experimental nanoindentation data with the Nix–Gao model’s predicted hardness values at depths below 150 nm, as the model tends to overestimate the density of geometrically necessary dislocations, potentially leading to higher modeled hardness compared to the actual measured nanoindentation hardness at very shallow depths. (31). In our experiments, the nanoindentation depths are much larger than 150 nm, and thus, some of these concerns are mitigated. The depth vs. time and velocity vs. time profiles of nanoindentation experiments at both quasistatic and dynamic strain rate regimes are depicted in Figs. S1a–f and S2a–f, respectively. The corresponding strain rate and load profiles have also been indicated in these figure panels. Furthermore, we show the hardness vs. depth profiles for both Z5 solutionized and peak-aged at different strain rates in Fig. S3a–d. The average and SD for hardness values were calculated between a depth of 2,200 and 3,000 nm. All data from these experiments are listed in Tables S4–S6.

In the quasistatic strain-rate regime ($10^{-3}\;s^{-1}$ to $10^{0}\;s^{-1}$), the average strength of the Z5 solutionized sample is much lower than the Z5 peak-aged sample (254 vs. 301 MPa, respectively). This difference in strength where the Z5 peak-aged sample continues to be stronger compared to the Z5 solutionized sample persists in the medium strain-rate regime ($10^{+1}\;s^{-1}$ to $10^{+2}\;s^{-1}$). However, the strength vs. strain-rate behavior begins to converge in the dynamic strain-rate regime ($10^{+3}\;s^{-1}$ to $10^{+4}\;s^{-1}$) with both samples reporting nearly similar strength values, 355 vs. 363 MPa for Z5 solutionized and Z5 peak-aged sample, respectively.

### Mechanisms related to quasistatic nanoindentation

At quasistatic and low values of strain rates ($10^{-3}\;s^{-1}$ to $10^{0}\;s^{-1}$), peak-aged Z5 is stronger than solutionized Z5. In the case of the peak-aged sample, finely distributed precipitates produced during the heat treatment process cover all the grains. When the indenter plunges into the peak-aged samples, the array of dislocations produced during deformation has to overcome these spatially distributed obstacles.

The critical resolved shear stress (CRSS) for doing so can be predicted as $\Delta \tau_{\mathrm{Orowan}}=G_{m}b/L$, where $G_{m}$ is the shear modulus of the matrix, *b* is the Burgers vector, and *L* is the spacing between precipitates (32). A further modification (33) to capture the dependence of the geometric configuration of precipitates can be written as


(1) $$\Delta \tau_{\mathrm{Orowan}}=\frac{G_{m}b}{2\pi (d_{s}-2r_{p})\sqrt{1-\nu }}\;\text{ln}\frac{2r_{p}}{r_{0}},$$

where $r_{0}$ is the core radius of the dislocation (34), $r_{p}$ is the average radius of the precipitates on the slip plane, *ν* is Poisson’s ratio, $d_{s}$ is the spacing between the precipitates on the glide plane, and $d_{s}=n_{s}^{-1/2}$, where $n_{s}$ denotes the number of precipitates per unit area. For the Mg system, one can calculate the effective planar, interprecipitate spacing $\lambda_{e}=d_{s}-2r_{p}$ of different precipitate morphologies in the Hexagonal Close-Packed (HCP) system and estimate the change in $\tau_{\mathrm{Orowan}}$ using Eq. 1. Let *f* denote the volume fraction of the precipitates, and assume that $r_{0}=b$. The Orowan CRSS from the *c*-axis precipitate rods present in the Mg–Zn alloy system is given by


(2) $$\Delta \tau_{\mathrm{Orowan},c}=\frac{G_{m}b}{2\pi \left[\frac{0.953}{\sqrt{\;f}}-1\right]d_{t}\sqrt{1-\nu }}\;\text{ln}\frac{d_{t}}{b},$$

where $d_{t}$ is the precipitate rod diameter (35). It follows from above that $\Delta \tau_{\mathrm{Orowan},c}$ depends strongly on *f* and precipitate diameter ($d_{t}$), which in turn depend on the effective interparticle spacing $\lambda_{e}$. From this equation, one can estimate the dependence of Orowon CRSS for any precipitate size and a given volume fraction (35). The Orowon increment in CRSS from Eq. 2 for the peak-aged Z5 alloy gives a value of $\Delta \tau_{\mathrm{Orowan},c}∼43$ MPa.

In the solutionized Z5, the alloying elements provide strengthening ($\Delta \tau_{\mathrm{ss}}$) via distortion of the lattice, which may be estimated as


(3) $$\Delta \tau_{\mathrm{ss}}=B_{r}c^{2/3}+B_{s}c^{2}{\left(1-c\right)}^{2},$$

where c=5% denotes the nominal concentration of Zn, $B_{r}=43.2$ MPa is the coefficient of random solid solution strengthening, and $B_{s}=6$ GPa is the coefficient of strengthening associated with short-range order (36). According to Eq. 3, $\Delta \tau_{\mathrm{ss}}{|}_{c=5\%}∼19$ MPa, implying that the Zn alloying content is roughly twice as effective in precipitate form as compared to solution form. It is worth noting that peak-aged Z5 has c∼1% concentration of Zn that remains in solution, which results in a small solid solution strengthening of $\Delta \tau_{\mathrm{ss}}{|}_{c=1\%}∼2$ MPa, according to Eq. 3. The difference in the quasistatic yield strength $\Delta \sigma_{Y}$ can be estimated via the Taylor factor, i.e. $\Delta \sigma_{Y}=M_{P-A}(\Delta \tau_{\mathrm{Orowan},c}+\Delta \tau_{\mathrm{ss}}{|}_{c=1\%})-M_{S}\Delta \tau_{\mathrm{ss}}{|}_{c=5\%}$, where $M_{S}∼4.5$ and $M_{P-A}∼3$ denote the Taylor factors for random solutionized Z5 and weakly textured peak-aged Z5, respectively. Substituting all the values, $\Delta \sigma_{Y}∼50$ MPa. This value is in good agreement with the experimentally measured difference in the average yield strength between the solutionized and peak-aged samples from nanoindentation experiments of ∼47 MPa and as shown in Fig. 2f.

### Mechanisms related to high strain-rate nanoindentation

Zerilli and Armstrong (37, 38) developed a constitutive model to characterize the strain-, strain rate-, and temperature-dependent response of HCP metals, by combining the terms from their earlier Body-Centered Cubic (BCC) and Face-Centered Cubic (FCC) constitutive models (39). Specifically, Zerilli and Armstrong concluded that overcoming Peierls–Nabarro barriers, associated with dislocation motion, was the principal thermal activation mechanism for BCC metals, whereas dislocation interactions, and thus density, were the governing mechanism for FCC metals. Further, they considered HCP constitutive behavior to combine mechanisms of both BCC and FCC strain-rate sensitivity. The current solutionized and peak-aged Z5 samples do, in fact, exhibit such a “BCC response”, that is, a rate-dependent initial yield strength commonly observed in BCC alloys. To this end, we employ the BCC term of the Zerilli–Armstrong (37) constitutive model for the yield strength, which is given by


(4) $$\sigma_{Y}=\sigma_{G}+\frac{k}{\sqrt{l}}+B\text{exp}\left[-\beta_{0}T+\beta_{1}T\ln\;\frac{\dot{\epsilon }}{{\dot{\epsilon }}_{0}}\right],$$

where $\sigma_{Y}$ is the yield strength, *l* is average grain diameter, *T* is the absolute temperature, $\dot{\epsilon }$ is the strain rate, and ${\dot{\epsilon }}_{0}$ is the reference strain rate. The model parameters are $\sigma_{G}$, the quasistatic strength due to alloying content and preexisting dislocations; *k*, the Hall–Petch slope; *B*, the strain-rate hardening modulus; $\beta_{0}$, the thermal-softening coefficient; and $\beta_{1}$, the rate-sensitivity parameter.

The total quasistatic strength $\sigma_{Y0}=\sigma_{G}+k/\sqrt{l}$ was fixed at the value obtained from the nanoindentation experiments. The other constitutive parameters required by the Zerilli–Armstrong model (Eq. 4) were fit to the experimental results of dynamic strength using a nonlinear regression algorithm, and are reported in Table S1. The excellent agreement between the Zerilli–Armstrong model and the experimental data is shown in Fig. 2h, in support of the “BCC response” assumption made in the constitutive analysis. As expected, $\sigma_{G}$ of peak-aged Z5 is greater than solutionized Z5, because the Zn alloying content is more effective at strengthening in precipitate form as compared to solute form. Both peak-aged and solutionized Z5 are found to have the same $\beta_{0}$ and $\beta_{1}$. That said, the strain-rate hardening modulus *B* is found to be larger in solutionized Z5 as compared to peak-aged Z5. Given that *B* is correlated with viscosity, this finding would seem to indicate that the viscosity of peak-aged Z5 is lower than the solutionized Z5. One possible explanation for this is that significant dislocation bowing around precipitates in peak-aged Z5 results in rapid dislocation multiplication at early deformation. The higher mobile dislocation density then results in a lower viscosity, (40). Another contributing factor is that solute atoms often decrease the mobility of dislocations (and hence increase the overall viscosity), (41). This argument would also support our finding that solutionized Z5 seems to be more rate-sensitive than peak-aged Z5. As a result of this higher rate sensitivity, the dynamic strength for these two alloys diminishes with increasing strain rate. Extrapolating our calibrated Zerilli–Armstrong model, we expect the solutionized Z5 to exhibit higher strength than peak-aged Z5 at sufficiently high strain rates, e.g. $≳10^{5}\;s^{-1}$.

In addition to the Zerilli–Armstrong model, we also employed three other commonly used constitutive models to describe the dynamic strengths of the solutionized and peak-aged Z5, i.e. the standard Johnson-Cook model (42), the modified Johnson-Cook model with quadratic form proposed by Huh and Kang (43), and the Cowper–Symonds model (44) (see Fig. S4a–d). The standard Johnson-Cook model, assuming a linear relationship between the dynamic strength and the logarithm of the strain rate, fails to capture the inherent nonlinearity present in our dataset. Further, comparing the coefficients of determination $R^{2}$ of these fitting results, both the Zerilli–Armstrong and the Cowper–Symonds models are capable of fitting the dynamic strengths over a broad range of strain rates. Note that both these models express the strength as a power of the strain rate.

Very few studies have reported high strain-rate nanoindentation of Mg alloys, especially in the $10^{+3}$ s^−1^ to $10^{+7}$ s^−1^ regime. Researchers have tested pure Mg and dilute Mg alloys with nearly ∼ 2–3 μm grain sizes up to a $10^{+2}$ s^−1^ strain rate (11). The small grain sizes ensured that the primary deformation mode was dislocation glide rather than twinning (11). The strength at high strain rates was influenced by the solute type, consistent with the theories of solid solution strengthening. Another study tested much coarser grained Mg alloys with ∼100 μm grain sizes at strain rates up to $10^{+2}$ s^−1^ (45). In this case, twins were found to form during the early stages of nanoindentation, resulting in strain compatibility constraints leading to cross-slip promotion within the characteristic activation volume. Another study found that the yield point in nanoscale indents was often identified by pop-in events, which had a strong rate dependence and much lower activation volume (46). In our study, given the large grain sizes, it is reasonable to assume that twinning is activated during the early stages of deformation indentation. Since the kinetics of twins and dislocations are closely related, using a pseudoslip approach to model twins is reasonable. Therefore, the Zerilli–Armstrong model is appropriate for solutionized and peak-aged Z5 whose plasticity is mediated by either dislocations, twins, or a combination of both, although it was developed initially based on dislocation motion. Unlike loading the material in pure tension or compression, the plastic strain field in the sample upon indentation is heterogeneous and should activate some twinning in every case, thereby reducing the effect of twinning on the polarity of stresses. Furthermore, in the case of Mg alloys, extension twinning itself is relatively strain-rate insensitive (47), even over the range of strain rates observed; therefore, the factor governing the strain-rate sensitivity is the dislocation-mediated plasticity. At low strain rates, strength is influenced by dislocation mobility, which in turn depends on the waiting time for dislocations to thermally overcome obstacles. At high strain rates, strength strongly depends on the number of mobile dislocations that heterogeneously nucleate from dislocation sources such as precipitates. In the solutionized sample at very high strain rates, every heterogeneous dislocation source will be tapped out, raising the strength to that of the peak-aged material. This is consistent with our experimental observations where the dynamic strength of the solutionized Z5 sample rises to that of the peak-aged Z5 sample toward the higher end of the dynamic strain-rate regime.

### Spall results—mechanical properties and microscopy

Plate impact experiments to induce and study spall behavior are a firmly established technique. Here, the internal material response is linked to rear surface displacements through well-established theory (1, 48). In these experiments, samples undergo a state of uniaxial strain to induce high tension within the material, facilitating void nucleation and failure. Upon void nucleation, a recompression wave is emitted and recorded on the rear surface. The wave propagation and interactions are illustrated through a Lagrangian time vs. position diagram in Fig. 1g. The plane waves generated in plate impact experiments can be viewed as the result of the Huyghens construction from an infinite array of identical point sources. In real materials, heterogeneities may generate signals that are slightly different from surrounding regions, and the contributions of such signals are shown in the diagram. The fully spalled samples experience approximately planar separation within the material, parallel to the wavefront imposed by the flyer plate loading as a result of the dynamic tensile stresses in this plane. The initially compressive shock stress ($\Sigma_{S}$) generated in the target material from the loading is calculated from


(5) $$\Sigma_{S}=\frac{1}{2}\rho_{0}U_{S}U_{B},$$

where $\rho_{0}$ is the reference density, $U_{S}$ is the shock speed estimated by assuming the linear equation of state with a parameter, $S_{1}$, of 1.21 for the AZ31B Mg alloy from Marsh (49) and $U_{B}$ is the rear surface velocity at maximum compression. The spall strengths of Z5 solutionized and Z5 peak-aged materials are obtained from the measured rear surface particle velocities using the following relationship:


(6) $$\Sigma^{*}=\frac{1}{2}\rho_{0}C_{0}(\Delta U_{fs}+\delta ),$$

where $\Sigma^{*}$ represents the spall strength, $\Delta U_{fs}$ is the velocity drop seen in Fig. 1h, $C_{0}$ is the bulk wave speed and *δ* is the elastic–plastic correction factor. The bulk wave speed was assumed to be 4,540 m/s, the reference density as 1,780 kg/m^3^, and the elastic–plastic correction factor is set to zero (17). The tensile strain rate, $\dot{\epsilon }$, is approximated via the velocity gradient by


(7) $$\dot{\epsilon }=\frac{1}{2C_{0}}\frac{\Delta U_{fs}}{\left|t_{c}-t_{d}\right|},$$

where $t_{c}$ and $t_{d}$ are the times at points c and d in (Fig. 1h). Representative photonic Doppler velocimetry spectrograms describing the time–frequency response of the spall signal are shown in Fig. 1g. More detailed spectrograms can be seen in Fig. S6a and b along with a compilation of the free-surface velocity traces for all spall experiments. The calculated spall strength and strain-rate values are plotted in Fig. 2i. The tensile strain rates for both sample sets range from 10^+5^ to 10^+7^ s^−1^. The Z5 solutionized samples have an average spall strength of 1.44±0.14 GPa, while the Z5 peak-aged samples have an average spall strength of 1.41±0.35 GPa. Tables S2 and S3 provide a summary of sample thickness and spall results for the Z5 solutionized and Z5 peak-aged datasets, respectively. A t test analysis suggests that there is no statistically significant difference in the spall strength between the two datasets, yielding a *P*-value of 0.77 (Fig. S5d). To limit the effect of strain rate dependency, which is amplified by the mismatched strain rate range between the datasets, we also perform the t test on a subset of data, from $9.42\times 10^{+5}$ to $1.44\times 10^{+6}$ s^−1^, where both material preparations have a significant overlap in the volume of experiments. The same analyses show little change in mean and median in the subset of the experimental data. Representative images from high-speed photography for the spall experiments are shown in the left columns of Fig. 3b and c. The postmortem SEM images of the 3 mm disks of both Z5 solutionized and Z5 peak-aged are shown in the right columns of Fig. 3b and c. For these spall experiments, the variation in $\Delta U_{\mathrm{fs}}$ as a function of peak shock stress is shown in Fig. S5a.

To see the trends in strength clearly, we also plot normalized effective stress (the ratio of shock stress to quasistatic yield strength) as a function of strain rate as shown in Fig. S5b (50). The quasistatic yield strength used for this ratio was taken from the nanoindentation experiments. From analyzing the spall results, we see that the spall strengths are nominally the same between the solutionized and peak-aged samples, with the solutionized microstructure perhaps exhibiting a slightly higher spall strength. However, the fracture and subsequent failure of the samples from the spall are dramatically different. We see that the damage is much more significant in the case of the Z5 peak-aged samples, per SEM micrographs shown in Fig. 3b and c, likely from precipitate-mediated void nucleation and coalescence.

The sharp contrast between the measured spall strength and the fracture morphology highlights the importance of not relying on the spall strength value alone to gain a complete understanding of the failure process of a material undergoing spall. The large number of experiments in this work is necessary to confidently identify these trends that might otherwise be lost through traditional low-throughput methods. While the measured spall strengths are similar, the fracture surfaces are dramatically different. In high strain-rate applications, such as for protection materials, knowledge of both the developed stress state and the postmortem failure morphology are crucial to understand material failure.

### Mechanisms related to spall

Our diagnostics provide both qualitative and quantitative understanding of the spall failure phenomena through imaging and in situ velocimetry, respectively, yet the imaging techniques show the greatest difference in the failure process in our experiments. The high-speed video and postmortem microscopy, shown in Fig. 3b and c, indicate that the spalled layer has completely fragmented away from the sample in the peak-aged Z5 case. In contrast, solutionized Z5 shows incomplete fragmentation in the spalled layer, more akin to a bulging separation.

The observed dichotomy in the failure mechanism (Fig. 3) is likely driven by defects or heterogeneities in the microstructure of the metallic alloy. Assuming that the damage during spall primarily nucleates (i) along grain boundaries or (ii) at precipitate–matrix interfaces, we can estimate differences in the density of sites for damage via void nucleation. We assume that the critical pressure for void nucleation sites *N* follows a bounded probability distribution function with a power-law exponent of β=3. Following the Wilkerson–Ramesh spall model (51), we assume that the density of potential nucleation sites along grain boundaries scales inversely with grain size *l*, and (following similar scaling arguments) that nucleation sites at second phase particles scale with the inverse cube of their mean spacing $d_{s}$, akin to volume fraction, i.e.


(8) $$N(l,d_{s})=N_{1}\left(\frac{l_{0}}{l}\right)+N_{2}{\left(\frac{d_{s}^{0}}{d_{s}}\right)}^{3},$$

where $N_{1}=1\;\mu {\text{m}}^{-3}$ and $N_{2}=10\;\mu {\text{m}}^{-3}$ are the densities of grain boundary and particle nucleation sites for a reference grain size of $l_{0}=1\;\mu \text{m}$, and reference mean particle spacing of $d_{s}^{0}=10$ nm, respectively. For peak-aged Z5, l=227μm and the mean particle spacing is equal to the precipitate spacing, nominally $d_{s}=50$ nm. For solutionized Z5, l=205μm and the mean particle spacing is taken to be a relatively large value governed by impurity content, $d_{s}=1\;\mu \text{m}$. The lower bound of the probability distribution function for the critical nucleation pressure is assumed to be a third of the limit critical tensile pressure of an idealized elastic-perfectly plastic material containing an infinitely small preexisting void, i.e.


(9) $$R_{y}\equiv \frac{2}{3}\left(\sigma_{G}+\frac{k}{\sqrt{l}}\right)\left[1-\ln\;\frac{3}{2}\left(\frac{\sigma_{G}}{E}+\frac{k}{E\sqrt{l}}\right)\right],$$

with E=47.4 GPa. This lower bound $R_{y}$ uses the $\sigma_{G}$ and *k* values taken from the nanoindentation data via the calibrated parameters from the aforementioned Zerilli–Armstrong model shown in Eq. 4, and governs the rate-independent spall strength (the rate-dependent contribution is discussed in more detail by Wilkerson and Ramesh (51)). The upper bound of the probability distribution function is taken as $R_{\mathrm{eos}}=7$ GPa, corresponding to the ideal spall strength of a perfect Mg crystal. The solid lines in Fig. 2j are model predictions of spall strength according to the Wilkerson–Ramesh spall model (51) invoking the aforementioned model parameters for solutionized and peak-aged Z5. Considering the experimental variability, the agreement between the model and experiments is remarkable.

Fig. S5c shows the theoretical predictions of the mean spacing between nucleated voids (dimples observed postmortem) on the spall surface of solutionized and peak-aged Z5 as a function of the experimentally measured spall strength. The dimples on the fracture surface of peak-aged Z5 are expected to be smaller by approximately a factor of 3 than for solutionized Z5. As such, the areal density of dimples on fracture surfaces is anticipated to be roughly a factor of 10 higher on peak-aged Z5 than solutionized Z5, so fractures linking the nucleated voids are expected to be significantly more prevalent in the peak-aged Z5.

While this model slightly overpredicts the difference in spall strength between the solutionized and peak-aged alloys, when compared to the averages from our laser-shock experiments, the model does offer a compelling explanation for the more brittle-like failure and complete separation of the spalled region, as observed in peak-aged Z5 (Fig. 3). The model curves shown in (Fig. 2j) employ a single set of nominal (average) values for precipitate spacing and grain boundary densities. As such, the model predictions are deterministic and make no attempt to capture the stochasticity observed in our dynamic strength or spall strength measurements (Fig. 2g–j). The stochasticity is expected due to the fact that both nanoindentation and LDMF plate experiments probe local, relatively small volumes of material, which may not contain a statistically representative feature of microstructure (e.g. texture). Moreover, variations in geometric morphology of the precipitates may contribute to the observed stochasticity, and are not captured by the model. As such, the locally measured properties are themselves spatially dependent for any type of experiment that does not probe a statistically representative volume of material. Interestingly, peak-aged Z5 microstructure (Fig. 2c and d) appears to exhibit significantly more spatial variability than the solutionized Z5 microstructure (Fig. 2a and b), which would seem to suggest that it would exhibit greater spatial variability in properties. Indeed, our spall strength measurements (conducted at various locations in the target plate) show significantly higher (spatial) variability in the peak-aged Z5 as compared to the solutionized Z5 (Fig. S5d).

## Conclusions

In this work, we combined two powerful small-scale mechanical testing techniques, namely custom dynamic nanoindentation and LDMF shock, to study the mechanical behavior of Mg alloys across a large strain-rate regime ($10^{-3}$ to $10^{+7}$ s^−1^). To the best of our knowledge, this is the first study to combine these two techniques in a single characterization campaign to probe mechanical behaviour across ten orders of strain rates. Two key advantages of our proposed approach are its high-throughput nature and its reliance on relatively small quantities of material, both of which make our approach particularly amenable to the “Materials by Design” paradigm. Our proposed high-throughput characterization methodology is harnessed to provide a deeper scientific understanding of the dynamic behavior of two Mg alloys. We tested Z5 solutionized (no precipitates) and Z5 peak-aged (with precipitates) across quasistatic, dynamic, and spall regimes. We measured Orowon yield stress increments at quasistatic strain rates and applied the Zerilli and Armstrong constitutive model to better understand the dynamic strength values. As expected, we found that at low to medium strain rates (i.e. quasistatic and dynamic), the Z5 peak-aged sample had higher strength when compared to the Z5 solutionized sample. However, at higher strain rates approaching $10^{+4}$ s^−1^, the dynamic nanoindentation experiments showed converging strength for both peak-aged and solutionized Z5. This seems to suggest that solution strengthening is just as effective as precipitate strengthening at very high rates.

Similar to very high strain rate dynamic yield strength, we observed that spall strength at very high strain rates was not strongly influenced by the presence of precipitates in the peak-aged alloy. In contrast, our postmortem observations of the spalled samples showed very different fracture behavior between the solutionized and peak-aged samples. The Z5 peak-aged samples failed more catastrophically due to precipitate-mediated void nucleation and the resulting accelerated spall fracture. We adopted a void nucleation and dynamic growth model to better understand the differences between solutionized and peak-aged Z5 samples. The model suggests that spall strength is most affected by the material’s inherent resistance to void nucleation, whereas fracture morphology is most affected by the number and spacing of nucleated voids. Based on our findings, we conclude that the inherent resistance to void nucleation is fairly insensitive to precipitate spacing, while the spacing of nucleated voids is strongly correlated with precipitate spacing. This demonstrated the importance of not only relying on the measured physical quantity of spall strength, which is often the default in engineering design work at ultrahigh strain rates, but also examining the damage morphology for a more complete understanding of material failure in extreme environments. Experiments on the same material systems through traditional low-throughput methods would not have readily shown these interesting trends.

Furthermore, the quasistatic yield strength values from nanoindentation experiments (that match well with Orowan predictions) are used in both the Zerilli–Armstrong constitutive model to predict dynamic strength at low to high strain rates, and also with the Wilkerson–Ramesh model to predict spall strength at ultrahigh strain rates, resulting in an excellent match with experimental observations of the dynamic strength and spall strength in their respective strain-rate regimes. The underlying quasistatic yield strength, therefore, unifies both of the models used to understand the underlying physics in each regime. This study demonstrates the potential for using high-throughput techniques to quickly map the mechanical properties of various metallic alloys and aid in their rapid development for extreme environments. We note the challenges in testing microstructures across disparate length scales (Section 4.4) but posit that our testing approach of combining custom dynamic nanoindentation and LDMF shock is currently the best available solution to this complex problem. Our testing framework provides rich opportunities to study how heterogeneities on different length scales influence the quasistatic, dynamic, and spall strength of materials. Finally, our study highlights an important lesson in paying attention to how microstructures can fail differently despite having similar strength at very high strain rates.

## Materials and methods

### Thermomechanical processing and sample preparation

The Mg–5Zn (at%) alloy, referred to as Z5, was prepared by melting and mixing high purity Mg (99.97%, Grade II, US magnesium) and Zn (99.999%, Alfa Aesar) in an argon atmosphere and casting into bars. The bars were solutionized at $500^{\circ }$C for 25 h to remove any preexisting precipitates from the casting process. The bars were then cut into rectangular pieces of 20 mm × 10 mm × 5 mm. Some of these pieces were then peak-aged at $150^{\circ }$C for 99 h to disperse precipitates throughout the bulk.

The solutionized and peak-aged Z5 pieces were then mechanically polished for further characterization and testing. A field emission scanning electron microscope (Carl Zeiss Crossbeam 1,540 EsB FIB/SEM) was used for electron microscopy studies and EBSD scans. The SEM has an HKL EBSD system with the Channel 5 software for EBSD analysis. For STEM studies, thin foil specimens, including grain boundaries, were placed by a standard lift-out technique using a dual beam FIB/SEM, FEI Helios G4. An FEI Titan G2 80–200 STEM was used for annular dark-field imaging and diffraction studies.

### Low to high strain-rate nanoindentation experiments

Nanoindentation is a well-established method for measuring the basic mechanical properties of materials and provides many advantages to traditional hardness testing (52, 53). In this work, nanoindentation was performed using a custom nanoindentation system made by KLA (USA) that is capable of testing in both the quasistatic and dynamic strain-rate regimes (details of the custom instrumentation are outlined by Hackett et al. (54)). A diamond indenter tip with the three-sided Berkovich geometry was used for all indents. The schematic of the nanoindentation system equipped with both the quasistatic straining protocol and dynamic straining protocol is shown in (Fig. 1a and b). For the quasistatic indents, a constant strain rate was achieved by loading such that the ratio of loading rate to load ($\dot{P}/P$) was constant. With a constant $\dot{P}/P$, the indentation strain rate (${\dot{\varepsilon }}_{i}$ ) can be calculated as


(10) $${\dot{\varepsilon }}_{i}\equiv \frac{\dot{h}}{h}=\frac{1}{2}\left(\frac{\dot{P}}{P}-\frac{\dot{H}}{H}\right)$$

with velocity ($\dot{h}$), indentation depth (*h*), loading rate ($\dot{P}$), load (*P*), change in hardness over time ($\dot{H}$), and hardness (*H*) (55). When *H* is constant, $\dot{H}=0$ and Eq. (10) simplifies to


(11) $${\dot{\varepsilon }}_{i}\equiv \frac{\dot{h}}{h}=\frac{\dot{P}}{2P}.$$

Hardness is constant as a function of depth for the tested Mg alloys, so Eq. (11) can be used. Quasistatic indents were performed to a maximum load of 200 mN and at $\dot{P}/P$s of 0.02 s, 0.2 s, and 2.0 s^−1^. During loading, CSMs were taken by applying a 110 Hz oscillation to the load such that the root mean square amplitude of the oscillation was 10% of the load as prescribed by Phani et al. (56). Results for *H* and ${\dot{\varepsilon }}_{i}$ were averaged over an indentation depth of 500–2,500 nm. A linear least squares fit of contact depth vs. indentation depth was used to determine a value for the ratio of contact depth to depth ($h_{c}/h$) in each material. This fit results in an $h_{c}/h$ of 0.94 and 0.98 for the solutionized and peak-aged material, respectively. Optical images of each indent were taken to confirm the residual contact impression matched the results of the CSMs.

An impact testing approach was used for the high strain-rate indentation, as described by Phani et al. (57) and Hackett et al. (54). To account for the dynamic overload during impact, loads of 30 and 50 mN were used so the final load on the sample would be between 200 and 300 mN, reaching comparable indent sizes with the quasistatic testing. Due to the nature of the impact test, a constant $\dot{P}/P$ cannot be maintained. Thus ${\dot{\varepsilon }}_{i}$ during these impact tests is not constant, allowing a range of strain rates to be reported from each test. Additionally, CSM cannot be used to measure *H* as CSM has been shown to have problems at high strain rates (12), and the instrument cannot apply the oscillation required for CSM at a frequency suitable for the <1 ms impact test. Without CSM, hardness is calculated with


(12) $$h_{c}=\frac{h_{c}}{h}\times h,$$

(13) $$A_{c}=\sum_{n=0}^{5}C_{n}h_{c}^{2^{\left(1-n\right)}},$$

(14) $$H=P/A_{c},$$

where $h_{c}/h$ was first determined during the quasistatic testing, and $C_{n}$ are a series of constants that describe the tip shape (52, 54). However, optical imaging of the residual contact impressions showed that the calculated contact area ($A_{c}$) when using $h_{c}/h$ from quasistatic testing did not align with the physical size of the indent. A new $h_{c}/h$ was calculated, 0.90 and 1.0, for solutionized and peak-aged, respectively, so that $A_{c}$ aligned with the measured contact area for the dynamic indents. A miniature ICP force sensor from PCB Piezoelectronics (USA) was added to the system to measure the applied load during dynamic testing. The addition of the piezoelectric force sensor lowers the instrument frame stiffness from 25 to 8 MN/m, but a standard frame stiffness correction can account for this correctly (52). Hardness and indentation strain were binned into 120 half-open bins between strain rates of $10^{1}$ and $10^{4}\;s^{-1}$.

### LDMF shock experiments

Laser-shock methods are well poised for high-throughput dynamic experiments; in contrast to conventional methods, these methods attain similar energy densities while operating more safely, at smaller scales requiring significantly less material, and at much lower overall expense (17, 58–61). The LDMF shock experimental setup, as shown in (Fig. 1) is a subset of these laser-shock methods that utilizes the energy from a laser pulse to accelerate a microscale flyer plate to achieve a high-velocity impact with a target, achieving tensile strain rates of ≥O(10^6^) s^−1^ during spall. We determine the dynamic response of the material through PDV measurements on the target’s free surface during the impact (17, 62). In contrast to traditional plate impact experiments, which average ∼ 1–3 experiments per day, LDMFs can obtain similar impact energy densities and can easily exceed 100 experiments per day. While the small scale of the LDMF experiment significantly reduces material waste, it requires careful consideration of the microstructure and deformation length scales at play. Even with recent attention to several shock compression applications (61), very few investigations are optimized to study spall failure. Recently, we have developed an LDMF system and methodology for investigating spall failure with high-throughput while maintaining reasonably high fidelity. In this effort, there are two critical challenges for establishing confidence and reproducibility: (i) maintaining a high degree of flyer planarity from launch to impact and (ii) establishing conditions such that a consistently high-quality PDV signal is obtained. This work is among the first experimental demonstrations (63) of our new methods to address these challenges.

The LDMF experiment consists of three sections: (i) the pulse laser and free-space optics; (ii) the impact setup; and (iii) the PDV diagnostics (Fig. 1e). The goal of the first section is to emit and manipulate the energetic, spatial, and temporal characteristics of the driving laser pulse to achieve optimal launch conditions (i.e. planar flyer at a desired velocity). Our system uses a 1,064 nm Nd: YAG 2.5 J 10 Hz 10 ns Spectra-Physics Quanta-Ray 350 with a beam quality (M2 value) of 15. The pulse energy drives the flyer velocity through the laser fluence. The pulse duration must be sufficiently long relative to the round-trip shock wave time inside the flyer to prevent reverberations during the launch that can break up the flyer (60). We employ an optical cavity to lengthen the pulse duration from ∼10 to ∼21 ns, which is sufficient for the flyer material and thickness used in these studies. Lastly, and perhaps most critical, the acceleration of a planar and intact flyer requires a homogenized beam profile, and so the beam is shaped just before the flyer launch. We utilize a custom-built diffractive optical element (DOE) by Silios Technologies to shape the beam profile to a low variation top-hat profile. This DOE is used in series with a focusing lens to shape the beam to a desired diameter at the effective focal length (EFL). A beam profiler is used to measure and monitor the beam shape during each experiment.

The second section of the experiment is the impact setup, which refers to the arrangement of the flyers and targets (Fig. 1f). Each impact setup has lateral dimensions of 50 mm by 50 mm and contains an array of strategically spaced flyers: here, a 7×7 square array yielding 49 experiments per package. Multiple launch packages are fabricated in advance, and the spall experiments are performed systematically through the prefabricated launch packages to improve experimental throughput. The design of the impact setup is critical for consistently and reliably achieving planar impact and strong PDV return signals at high-throughput rates. It is a layered structure foil as shown in Fig. 1f and consists of a substrate, a flyer, a spacer, and a target. The glass substrates are 50 mm × 50 mm × 0.625 mm borosilicate glass from McMaster Carr, and the epoxy used for bonding is Henkel Loctite Ablestik 24. The flyers are 100 μm thick, 1.5 mm diameter aluminum from Alufoil, and the spacer is a 240-μm thick Kapton sheet with built-in double-sided silicone-based adhesive. The targets (samples) are prepared through double-sided polishing of larger area foils to a thickness of 200± 10 μm, and then 3 mm disks are created using a TEM-punch (19). The flyer and target thickness are determined based on wave propagation analysis so that the spall plane occurs within the target. Their diameters are sufficiently large to avoid any unloading wave effects on the central area of interest during the time of interest. The flyers are precut using a femtosecond laser to obtain a flyer with a sufficiently large diameter in order to prevent edge unloading from affecting the PDV signal and to guarantee impact planarity. The pulse laser is operated at ∼ 800 mJ and is focused to a spot size of 1.85 mm with a 250 mm EFL. This yields fluences of ∼ 30 to 32 J cm^−2^ that drive impact velocities ∼550 to 600 ms^−1^.

The diagnostics consist of a high-speed camera and a PDV system. The high-speed camera (Shimadzu HPV-X) operates at 10 million frames per second. It provides a side profile view of the impact setup for qualitative information regarding the flyer and impact planarity and a macroscopic assessment of the developed spall damage. The PDV system measures the normal particle velocity of the rear free surface of the target (17, 62), as shown in Fig. S6. The PDV is a heterodyne system that consists of two 1,550 nm centered fiber lasers, a seed signal laser directed toward the target, and a 2.3 GHz upshifted reference signal for mixing. The seed laser is focused on the backside of the sample with a spot size of 80 μm; the material response is averaged over this area, so this length scale must be sufficiently large relative to the relevant material length scales. During the experiment, the reflected light (return signal) is imparted with a frequency shift based on the particle velocity. The return signal is mixed with the reference signal to obtain the beat frequency, which is measured and recorded using a 16-GHz LeCroy Oscilloscope. The strength of the return signal, and therefore the quality of the spall signal, is a strong function of the target’s surface roughness and orientation. We use a custom-built alignment apparatus that allows fine orientation control of the impact setup for optimizing the return signal. Samples are double-sided polished to high reflectivity with diamond lapping paper to a 1-μm mirror finish to further maximize the return signal.

### Limitations of the testing techniques

The two testing approaches proposed in this paper, namely custom dynamic nanoindentation and LDMF shock can result in testing across disparate length scales and care has to be taken while probing mechanisms or effective volumes under study. For example, if the materials being designed or tested have features, such as texture or grain size, that vary across 10 s of microns, there could be size effects that might ultimately influence the strength measurements. In the case of nanoindentation of Mg alloys, the plastic zone is often found to be an order of magnitude higher than the indentation depth (64, 65). Small grain size and twinning are found to strongly influence the deformation mechanisms during indentation (46). In our case, we note that the indentation depths range from 0.5 to 2.5 μm and the grain sizes are coarse (∼205 to 227 μm). In the case of spall, laser-shock experiments use small volumes of material in comparison to those relevant to many bulk applications, and so the results must be viewed with caution when applying them to some larger scale structures. Effectively, we are sampling small volumes out of a larger volume, and so spatial heterogeneities on scales larger than the laser-shock sample sizes have to be viewed from a statistical sampling perspective. If the relevant defect/feature distributions in the material are sufficiently small, the sampling statistics are less of an issue (17, 19, 63, 66, 67). We also note some limitations with the PDV approach used in spall experiments. The free-surface velocity signal at a point on the rear surface is composed of data from a domain in the material determined by the wave speeds (information at any time at that point relates to the state at other points in the volume at earlier times). The PDV measures the particle velocity history *v(x,t)* of a small part of the rear surface of the target—the PDV spot size is 80 μm, where *x* is the center of the spot, and it measures only surface motion. That surface velocity arises because of the arrival of a plane shock wave that has propagated through the target plate and that then interacts with the free surface to reflect as a rarefaction fan. The nature of the wave propagation is such that the particle velocity at any position *x* and any time *t* is the result of waves arriving at that point from all locations in the plate within a distance $U_{s}t$. Each of those waves carries information about the state of stress and deformation at that location. Given that the shock speed in such experiments is of the order of 6 km/s, at a time t=40 ns information has arrived at the PDV sensing location from points ∼240μm away, and thus the PDV is providing information from the bulk of the specimen after that time. However, we note at the earliest times in the experiment, smaller volumes are being interrogated.

In the present study, the testing volumes captured significant volume fractions of microstructure features of interest. In the case of solutionized Z5 samples, Zn atoms (r∼ 139 pm) are uniformly distributed in the metallic solid solution and there should be multiple millions of atoms inside 1 $\mu {\text{m}}^{3}$ of the alloy sample. In the case of peak-aged samples, the volumetric number density of nanoscale precipitates is ∼1,56,000 precipitates for 1 $\mu {\text{m}}^{3}$ of the alloy sample. Thus, there should be sufficient populations of microstructural features of interest (Zn atoms in the solutionized sample and MgZn precipitates in the peak-aged samples) in the testing volumes being probed by the two proposed experimental methods used in our paper. Consequently, our testing framework can help in the design of several advanced alloys that often have design features at the lower length scales, such as atomic-scale chemical heterogeneity, nanoscale precipitates, or oxides found in dispersion strengthened alloys. Furthermore, our proposed approach can be used to accelerate the design of several classes of metallic alloys by down-selecting alloys and guiding the design of microstructural features of interest, while large-scale experiments can be employed to probe material performance with larger samples, closer to those used in the application space. Combining small and large-scale testing appropriately can accelerate the design of metallic alloys for extreme dynamic environments in a high-throughput manner.

## Supplementary Material

pgae148_Supplementary_Data

## Data Availability

The datasets generated and codes used in the current study are publicly available at https://craedl.org/pubs?p=6352t=3c=187s=hemid=https:%2F%2Ffs.craedl.org#publications.

## Preprints

This manuscript was posted on a preprint: https://arxiv.org/abs/2309.14296.
