# Supplementary material for: High-throughput quantification of quasistatic, dynamic and spall strength of materials across 10 orders of strain rates
Source: PNAS Nexus. 2024 Apr 5;3(5):pgae148. doi: 10.1093/pnasnexus/pgae148 (PMC11231947; doi:10.1093/pnasnexus/pgae148)
Supplement: pgae148_Supplementary_Data [file pgae148_supplementary_data.pdf]

SI File: High-throughput quantification of dynamic and spall strength of materials across ten orders of strain rates

Suhas Eswarappa Prameela[a,b,c,d,*], Christopher C. Walker[e,m], Christopher S. DiMarco[d,f,m], Debjoy D. Mallick[d,g], Xingsheng Sun[h], Stephanie Hernandez[i], Taisuke Sasaki[j,k], Justin W. Wilkerson[e,l], K.T. Ramesh[d,i], George M. Pharr[e], Timothy P. Weihs[c,d]

[a]*Department of Materials Science and Engineering, MIT, Cambridge, MA, USA*
[b]*Department of Aeronautics and Astronautics, MIT, Cambridge, MA, USA*
[c]*Department of Materials Science and Engineering, Johns Hopkins University, Baltimore, MD 21218, USA*
[d]*Hopkins Extreme Materials Institute, Johns Hopkins University, Baltimore, MD, 21218, USA*
[e]*Department of Materials Science and Engineering, Texas A&M University, College Station, TX, 77843, USA*
[f]*Sindri Materials Corp., West Chester, PA, 19382, USA*
[g]*DEVCOM Army Research Laboratory, 321 Colleran Road, Aberdeen Proving Ground, MD, 21005-5066, USA*
[h]*Department of Mechanical and Aerospace Engineering, University of Kentucky, Lexington, KY 40506, USA*
[i]*Department of Mechanical Engineering, Johns Hopkins University, Baltimore, MD 21218, USA*
[j]*National Institute for Materials Science, Tsukuba 305-0047, Japan*
[k]*Center for Elements Strategy Initiative for Structural Materials (ESISM), Kyoto University, Kyoto 606-8501, Japan*
[l]*J. Mike Walker '66 Department of Mechanical Engineering, Texas A&M University, College Station, TX, 77843, USA*
[m]*These authors contributed equally to this manuscript*

---

*Corresponding author
Email address: suhasep@mit.edu (Suhas Eswarappa Prameela)

1. Supplementary Information

1.1. Details regarding the data from custom Nanoindentation set-up

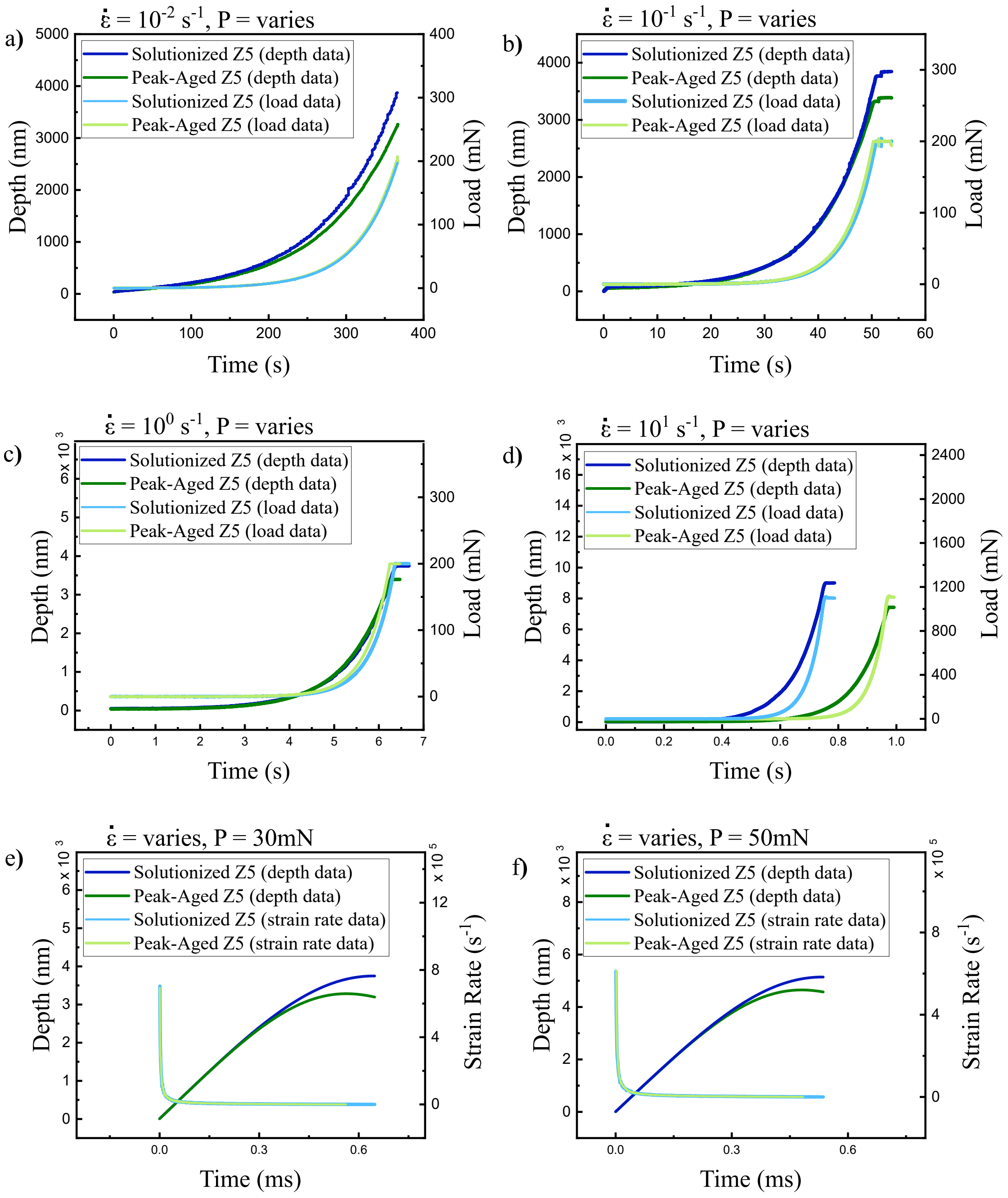

FS 1: Depth vs. time for nanoindentation tests at different strain rates. For quasistatic tests (a-d), the load vs. time profiles are given to show the exponential loading used. For impact indentation tests (e-f), strain rate vs. time profiles are shown to indicate the non-constant strain rate during loading.

FS 1 shows the exemplary depth-time and load-time data for the quasistatic nanoindentation testing. A 1 kHz feedback control loop was used to maintain a constant strain rate while loading to 200 mN. The difference between the final depths of the solutionized Z5 and Peak-Aged Z5 is a result of the small difference in hardness at each strain rate. The final load at $\dot{\epsilon} = 10^1\,s^{-1}$ is much larger than the target 200 mN due to dynamic effects brought on by the order of magnitude increase in velocity required to maintain the constant strain rate (seen in FS 2). Additionally, as the load is only updated in discrete steps every 1 ms by the control loop, the final load step is often larger than necessary

to reach the target load in very fast tests. FS 1 (e-f) shows depth-time and strain rate-time data for high strain rate impact nanoindentation tests. The strain rate is shown instead of load due to the strain rate not being constant throughout the experiment. Load is also not constant, but due to the nature of the impact test, a fixed impact force is given, and then most of the experiment is driven by dynamic forces from the rapid deceleration at the moment of impact.

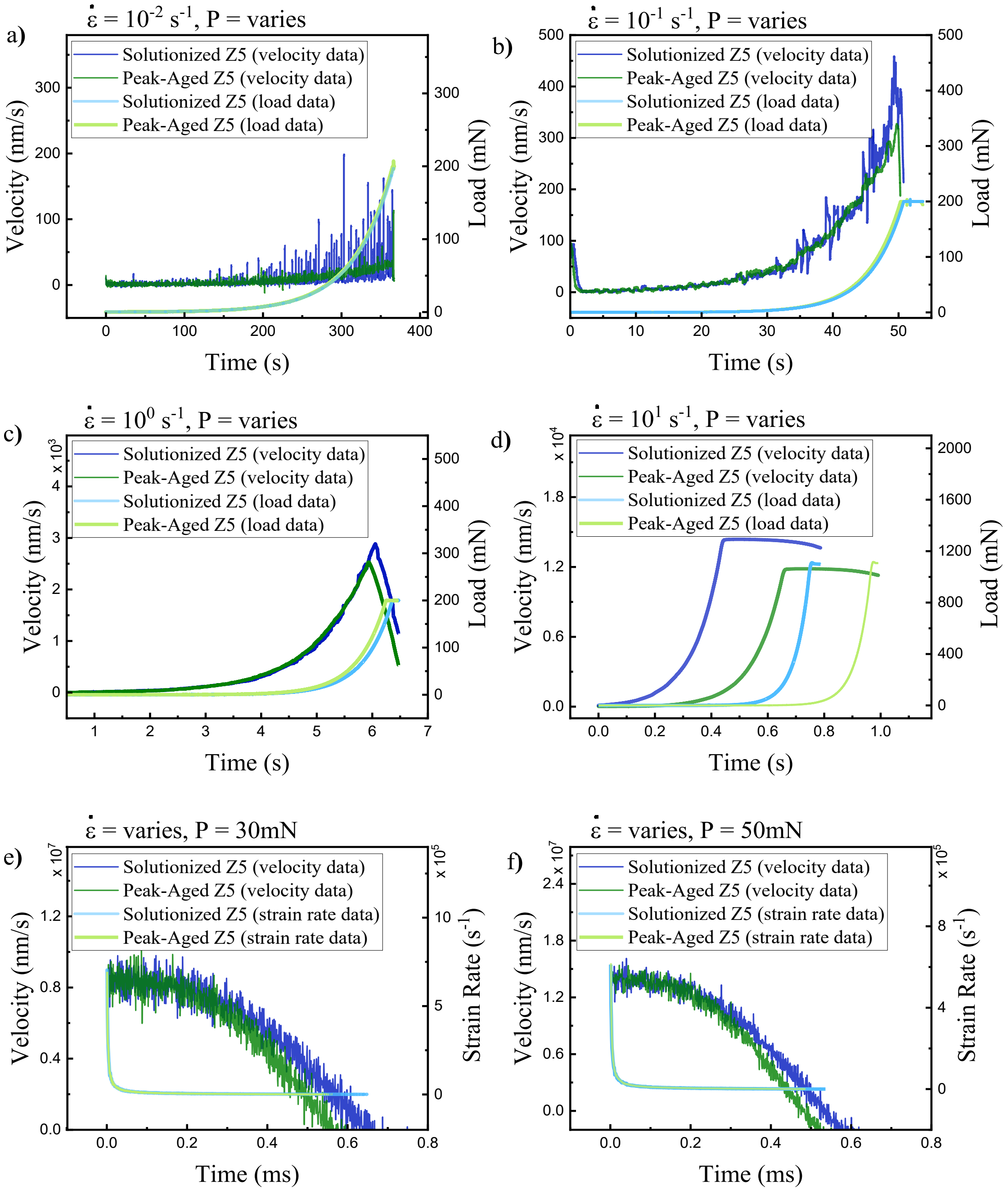

FS 2: Velocity vs. time for nanoindentation tests at different strain rates. For quasistatic tests (a-d), the load vs. time profiles are given to show the exponential loading used. For impact indentation tests (e-f), strain rate vs. time profiles are shown to indicate the non-constant strain rate during loading.

FS 2 highlights velocity vs. time for each of the corresponding tests in FS 1. The exponentially increasing velocity is necessary during quasistatic nanoindentation to maintain a constant strain rate as depth increases and, therefore contact area between the diamond tip and sample increases. The velocity at low strain rates ($10^{-2}\,\mathrm{s}^{-1}$ to $10^{-1}\,\mathrm{s}^{-1}$) is particularly noisy due to the relatively small velocity of the test compared to the large change in velocity from the constant stiffness measurements (CSM) oscillation. At $\dot{\epsilon} = 1\,\mathrm{s}^{-1}$ (FS 2 (c)) the velocity of the test is much larger than the velocity of

CSM, making the noise negligible. At a strain rate of $10^1\,\mathrm{s}^{-1}$ (FS 2 (d)), CSM cannot be used since the high velocities result in a very short test and the 1 kHz control loop produce a high enough oscillatory frequency to capture quality stiffness measurements while maintaining the increasing velocity. For $1\,\mathrm{s}^{-1}$ and $10^1\,\mathrm{s}^{-1}$, the decreasing and flat velocities (respectively) after reaching peak velocity are a result of the dynamic forces generated by the high velocities and accelerations and vary depending on the timing of the last force step and the amount of time it takes the feedback control loop to determine it is at or past the target load. For the impact tests in FS 2 (e-f), the test is designed so that contact with the sample surface is made at the instance of max velocity. No additional force is added to the system after this point to allow dynamic effects to drive the loading process. This results in a decreasing velocity throughout the entire experiment.

The measured hardness from both quasistatic and impact nanoindentation experiments are shown in FS 3. FS 3 (a-c) shows that hardness vs. depth is fairly constant for both the solutionized Z5 and peak-aged Z5 samples after some initial indentation size effect (ISE). To mitigate the effects of ISE on the reported hardness, an average hardness between 2200 nm and 3000 nm is reported in FS 3 (e). Hardness vs. depth is also reported for the impact nanoindentation experiments in FS 3 (d), which shows a decreasing hardness as a function of depth even past a depth where hardness is flat in the quasistatic results. This decrease in hardness is explained by FS 3 (f), which shows hardness decreases as the strain rate decreases. During a nanoindentation impact experiment, the strain rate is not constant and decreases until the very end of the test where it falls to effectively $0\,\mathrm{s}^{-1}$ at the peak load. Though strain rates as high as $5 \times 10^5\,\mathrm{s}^{-1}$ can be seen in FS 1 (e-f) and FS 2 (e-f), hardness is only reported for $\dot{\epsilon} \leq 10^4\,\mathrm{s}^{-1}$ to avoid edge effects created the strain rate being infinite at the moment of impact.

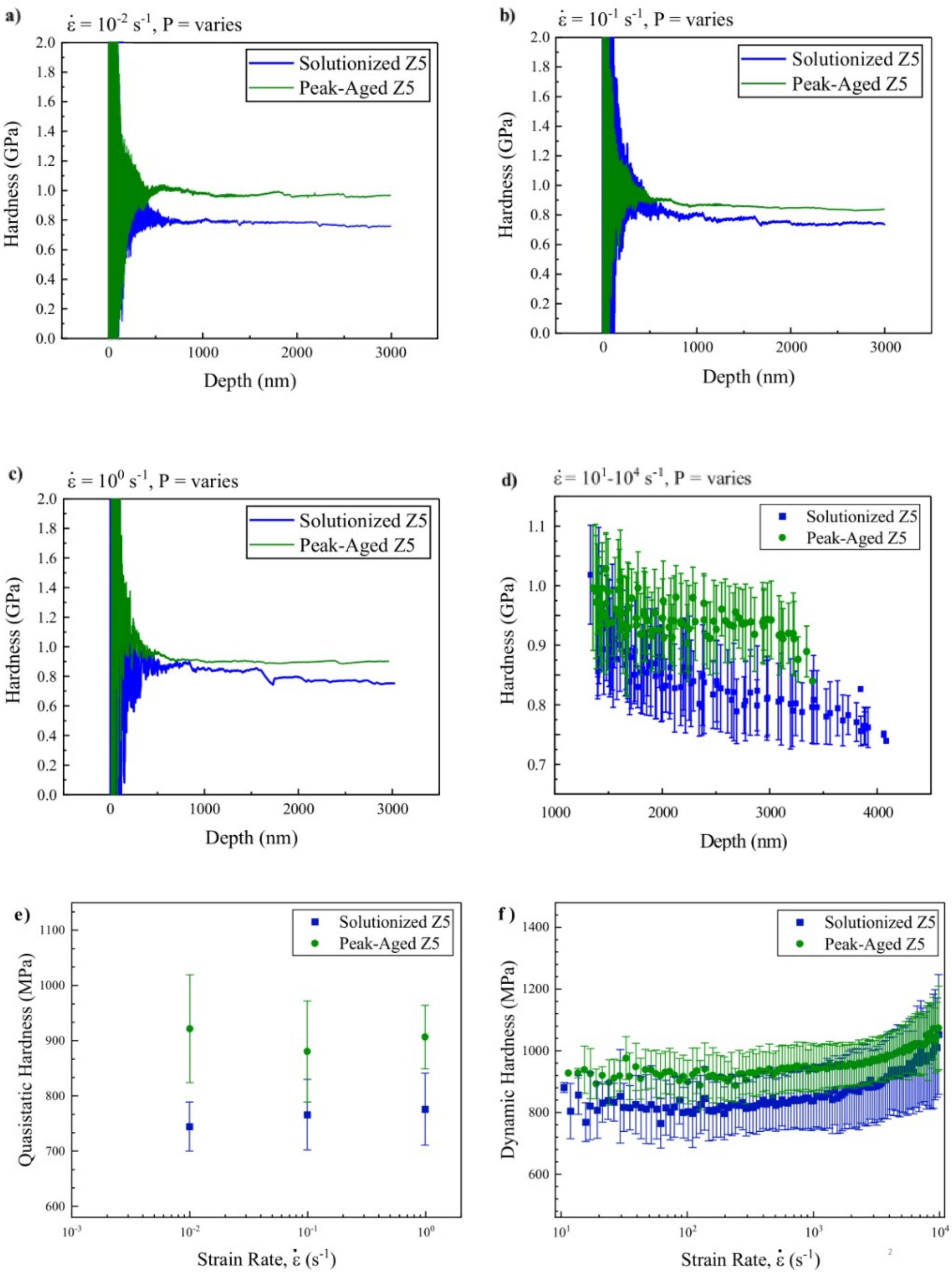

FS 3: The average and standard deviation for hardness values were calculated between a depth of 2200 nm and 3000 nm. A representative hardness vs. depth for nanoindentation tests for Z5 (wt%) solutionized and peak-aged at strain rate a) $10^{-2}s^{-1}$, b) $10^{-1}s^{-1}$, c) $10^{0}s^{-1}$and d) 10^{1} to $10^{4}s^{-1}$, e) Quasistatic hardness measured via nanoindentation, f) Dynamic hardness measured via nanoindentation.

1.2. Details regarding the strength modeling on the Nanoindentation data

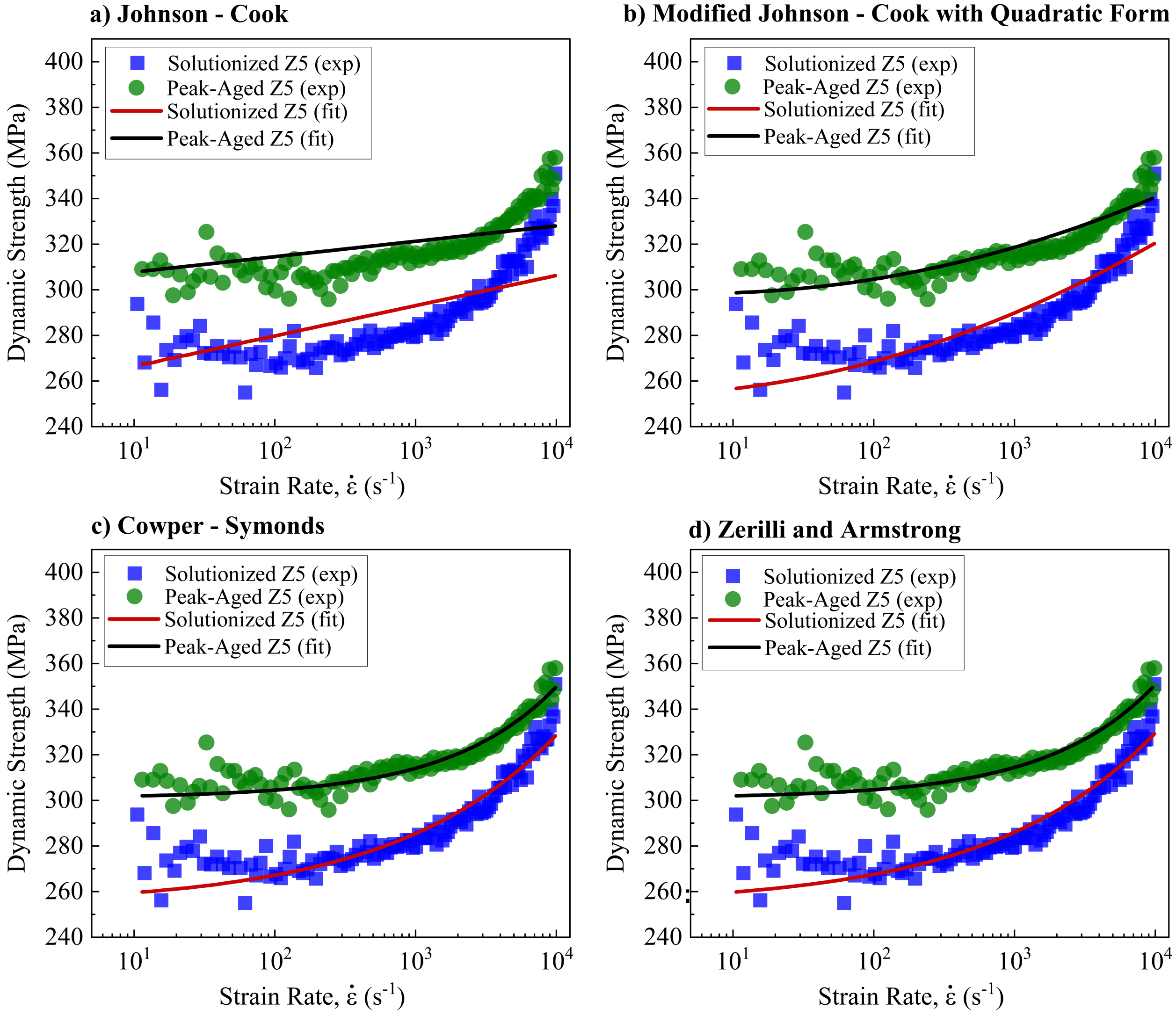

FS 4: Comparisons of dynamic strengths between nanoindentation measurements and constitutive model fittings for solutionized and peak-aged Z5 samples: a) JC model with R^2 of 0.58 for solutionized and 0.47 for peak-aged, b) JC-quad model with R^2 of 0.77 for solutionized and 0.78 for peak-aged, c) CS model with R^2 of 0.86 for solutionized and 0.89 for peak-aged, and d) ZA model with R^2 of 0.86 for solutionized and 0.89 for peak-aged.

In addition to the Zerilli-Armstrong (ZA) model, we also employ three other constitutive models to describe the dynamic strength of the solutionized and peak-aged Z5 depending upon strain rate, i.e., the standard Johnson-Cook (JC) model [1], the modified JC model with quadratic form (JC-quad) proposed by Huh and Kang [2], and the Cowper-Symonds (CS) model [3]. Specifically, the JC form of the yield strength at room temperature is written as Eq 1 [1]. In Eq 1, ϵ_0 is the reference strain rate. The fitting parameters are the quasistatic yield strength σ_{Y0} and the strengthening coefficient of strain rate C. The JC-quad form of the yield strength is given by Eq 2. In Eq 2, σ_{Y0}, C_1 and C_2 are fitting parameters. Moreover, the yield strength with the CS form can be expressed by Eq 3. In Eq 3 σ_{Y0}, D and P are fitting material constants.

$$\sigma_Y = \sigma_{Y0}\left(1 + C\ln\left(\frac{\dot{\epsilon}}{\dot{\epsilon}_0}\right)\right), \tag{1}$$

$$\sigma_Y = \sigma_{Y0}\left(1 + C_1 \ln\left(\frac{\dot{\epsilon}}{\dot{\epsilon}_0}\right) + C_2 \ln\left(\frac{\dot{\epsilon}}{\dot{\epsilon}_0}\right)^2\right), \tag{2}$$

$$\sigma_Y = \sigma_{Y0}\left(1 + \left(\frac{\dot{\epsilon}}{D}\right)^{1/P}\right), \tag{3}$$

The JC model, due to its simple formulation and clear physical meanings of the model parameters, is a widely used constitutive model for simulating the mechanical behavior of materials, especially in the context of high-strain-rate deformation, such as in impact or explosive loading scenarios. However, the standard JC model assumes a linear relationship between dynamic strength and the logarithm of the strain rate. The modification with a quadratic form, i.e., the JC-quad model, introduces additional parameters that enhance the strain-rate sensitivity of the model. This allows the model to better capture and represent the nonlinear behavior of materials under dynamic loading, which may be essential for accurate simulations. In addition, the CS plasticity model has a relatively simple mathematical formulation compared to more complex material models. This simplicity makes it computationally efficient and easy to implement in numerical simulations. The CS model also incorporates strain rate sensitivity, allowing for an improved representation of material behavior under dynamic loading conditions. A comparison of the fitting results with experimental data is shown in FS. 4. It is noteworthy that the quasistatic strength σ_{Y0} in all the constitutive models was fixed at the value obtained from the nanoindentation experiment. The fitting parameters are tabulated in Table S1. Detailed discussions of the fitting results are provided in the main text.

Table S1
Constitutive model parameters for dynamic strengths of solutionized and peak-aged Z5.

Parameter		Unit	Solutionized	Peak-aged
JC model	σ_{Y0}	MPa	254	301
	C	-	0.02242	0.009752
JC-quad model	σ_{Y0}	MPa	254	301
	C_1	-	-0.003581	-0.009356
	C_2	-	0.003477	0.002553
CS model	σ_{Y0}	MPa	254	301
	D	-	2.583×10^5	2.394×10^5
	P	-	2.683	1.747
ZA model	σ_{Y0}	MPa	254	301
	σ_G	MPa	217	266
	k	$\text{MPa} \cdot \mu\text{m}^{1/2}$	526	526
	B	MPa	2.4413	0.2510
	β_0	K^{-1}	0	0
	β_1	K^{-1}	0.0012	0.0019

1.3. Details regarding the data from custom laser spall set-up

All spall data results, including raw and analyzed PDV results, high-speed camera footage, experimental parameter data sheets, and Python-based PDV code, are available via the link in the Data and Code Availability section of the main text. A reference file in the main directory summarizes the results and maps them with their relevant PDV and high-speed camera data files. A FileMaker relational database was created to track metadata associated with the experimental methods and techniques, and this data was exported into Excel sheets for reference.

Selected high-speed camera videos were chosen to clearly demonstrate the custom laser-driven micro-flyer experiment and the spall failure results of the two data sets. The videos were recorded from a side angle, showing the flyer accelerating from top to bottom in the frame. During impact experiments, the flyer is not visible to the PDV or camera. Therefore, the flyer's velocity and planarity are determined independently before impacting a sample. The first video demonstrates the launch of a single flyer in the absence of a sample to showcase the high degree of planarity achieved. The second and third videos show characteristic impact experiments of solutionized Z5 samples, while the fourth and fifth videos show characteristic impacts for peak-aged Z5 samples. The high planarity maintained during the impact event indicates the planarity of the event itself. Solutionized samples demonstrate higher damage resistance

than peak-aged samples when impacted under the same conditions.

Video 1	Flyer Launch	Link 1
Video 2	Solutionized Z5 Impact 1	Link 2
Video 3	Solutionized Z5 Impact 2	Link 3
Video 4	Peak-Aged Z5 Impact 1	Link 4
Video 5	Peak-Aged Z5 Impact 2	Link 5

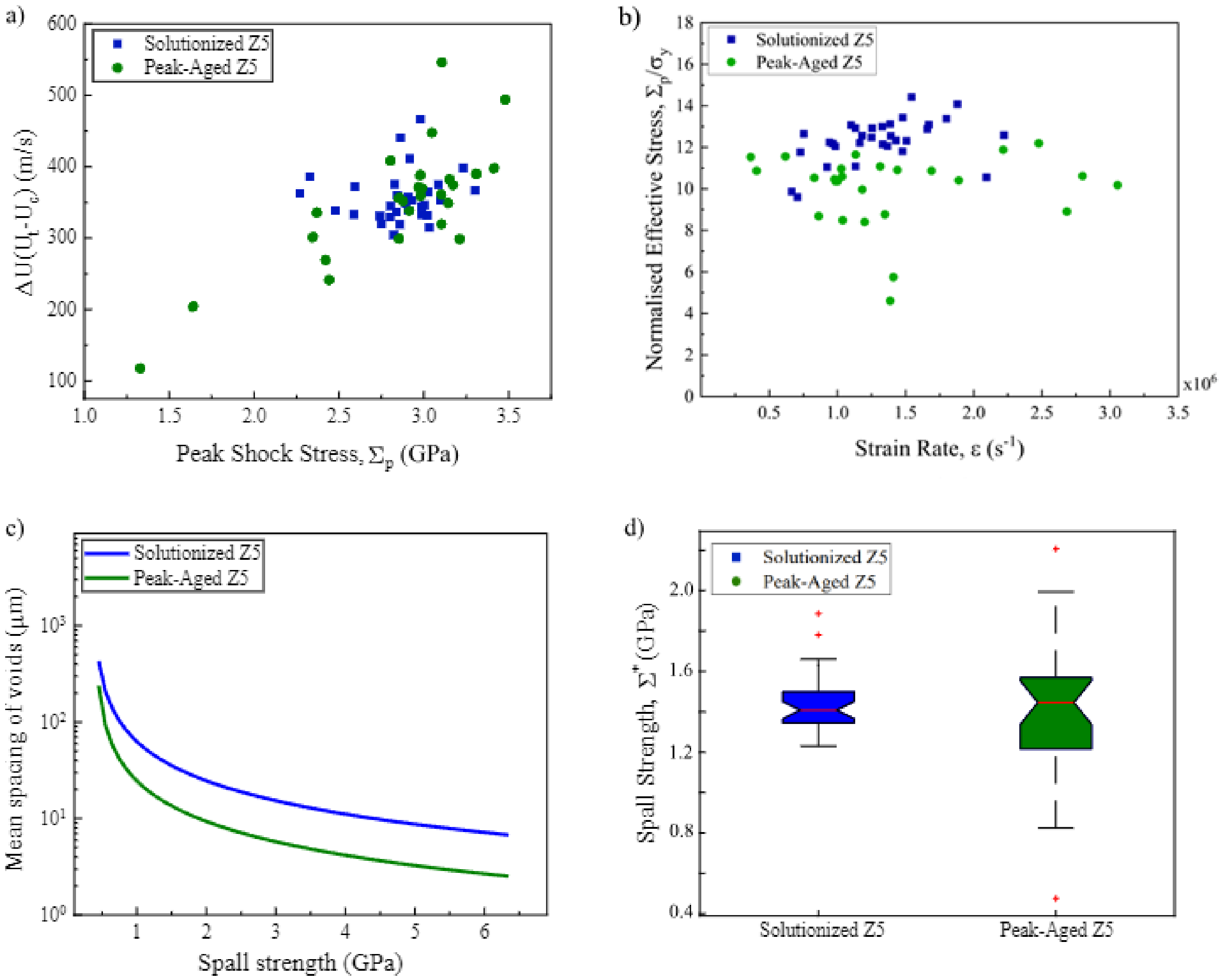

FS 5: a) Delta U vs. Shock Stress, b) Normalized Effective Stress vs. Strain rate, c) Mean spacing of voids vs. experimentally measured spall strength, d) Spall strength of solutionized Z5 and peak-aged Z5 samples.

The spall results are presented in two plots in FS 5. FS 5a displays the pullback velocity against the peak shock stress for both sample sets. While spall results are typically plotted against strain rate, it is not directly controllable in laser-driven micro-flyer experiments. Instead, the impact velocity is controlled, which determines the peak shock stress in the material. As peak shock stress increases, metals experience strain-hardening from the initial shock compression wave before spall failure. The correlation is clearly observable with the peak-aged samples, while the solutionized sample set is too narrow to draw definitive conclusions. The box plot in FS 5d provides a more rigorous statistical analysis and a t-test. An ANOVA test indicates that there are no statistically significant differences in the spall strengths between the two datasets within a 95% confidence interval, which is also evident from the comparison of the box-plot

distributions. In this plot, the box delineates the interquartile range, capturing the central 50% of the data between the lower and upper quartiles, with the median positioned at its narrowest part. The whiskers extend to the minimum and maximum data points, while plus sign markers highlight any outliers. FS 5d illustrates that the medians and interquartiles are well-aligned, indicating a relatively consistent distribution. Additionally, the solutionized samples exhibit a narrower distribution.

FS 5b plots the normalized effective stress against the strain rate, where the former is calculated as the peak shock stress divided by the yield strength. The yield strength of a material is a measure of its resistance to spall failure, with higher yield strength indicating higher resistance to void growth. In FS 5b, lower normalized effective stress corresponds to higher spall resistance. These results highlight the differences between the two datasets and are consistent with the observed variations in damage morphology. Specifically, the peak-aged samples exhibit a higher normalized effective stress and sustain more significant damage under the same impact conditions. FS 6 shows a summary of the PDV results for both datasets, with essentially the same information but for each dataset displayed in the top and bottom frames. Within each frame, the top four figures represent how our PDV code extracts the PDV trace and relevant data points. While a short-time Fourier transform is used for viewing purposes, the PDV code employs direct phase differentiation for more accurate analysis of high-frequency waves. First, the spectrum is imported and coarsely pre-filtered to include only the expected data range. Second, the starting point of the signal is identified, and a finer time-based filter is applied based on the expected duration of the signal. Third, the spall signal is isolated by filtering out the frequency upshifted signal. Fourth, the signal is differentiated, the velocity trace is calculated, and the critical data points are automatically determined. The fourth frame shows the identification of the velocities at maximum compression and tension. The PDV code, processing input parameters, and a graphical summary of results are included in the shared data directory. Lastly, at the bottom of each frame, a compilation of all PDV traces is presented side-by-side for an easily viewed summary and direct comparison.

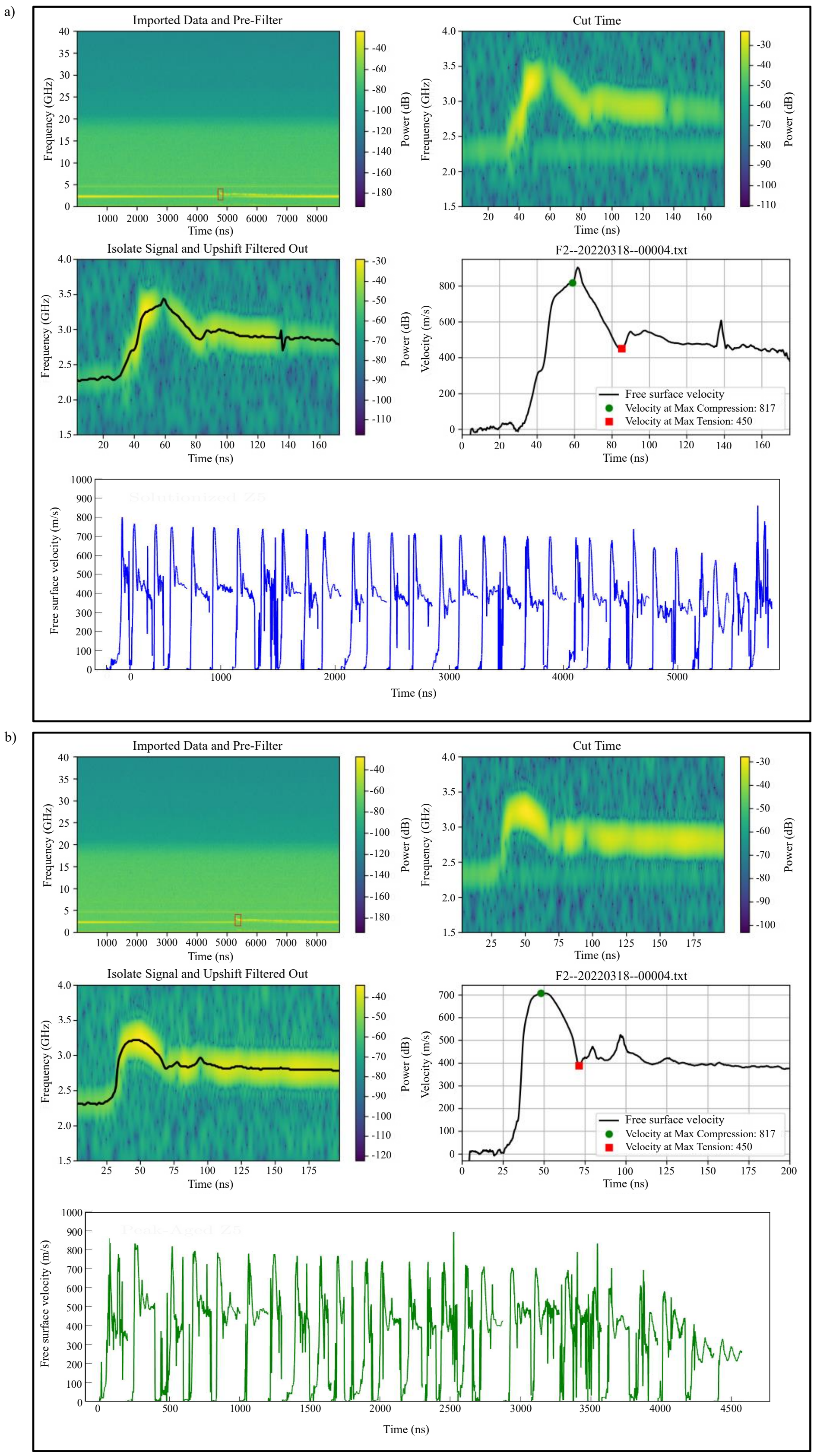

FS 6: Representative photon Doppler velocimetry spectrograms describing the time-frequency response and free surface velocity of the spall signal from a) solutionized Z5 and b) peak-aged Z5

Tables S2 and S3 provide a concise summary of the spall failure results for the solutionized and peak-aged samples, respectively. These tables include information such as the thickness of each individual sample, the peak shock stress, spall strength, and strain rate. The velocities at maximum compression and tension are also included in the shared data link. The thickness of each sample was measured using a micrometer prior to testing. The remaining quantities were determined based on the material density, the bulk wave speed, the equation of state, and the velocities at maximum compression and tension. These relationships are provided in the main text and were used to calculate the spall failure results for each sample.

FS 5c shows the theoretical predictions of the relationship between mean spacing of nucleated voids (dimples that might be observed postmortem) on the spall surface of the solutionized and peak-aged Z5 specimens as a function of the experimentally measured spall strength, per the analytic model by Wilkerson and Ramesh, 2016. In both the solutionized and peak-aged cases, the void spacing will decrease as the spall strength increases, but the increased density of failure nucleation sites in the peak-aged case will lead to smaller void spacing when compared to the solutionied case for a given spall strength, with the difference in spacing increasing as the spall strength increases. Future investigations are planned to further vary the peak aging conditions to obtain samples with different precipitate mean spacings and further establish the nature of precipitate interphase and its correlation to failure mechanisms.

Table S2
The results of spall experiments on solutionized Z5 alloy including thickness (μm), strain rate (s^{-1}), shock stress (GPa), spall strength (GPa) and pullback velocity (m/s).

Shot No.	Thickness (μm)	Strain rate (s^{-1})	Shock stress (GPa)	Spall strength (GPa)	Pullback (m/s)
1	214	1880410	3.58	1.61	398.47
2	211	1658727	3.27	1.89	466.90
3	207	2222355	3.20	1.66	411.15
4	215	2093194	2.68	1.37	338.79
5	213	1428659	3.13	1.78	440.73
6	201	1367488	3.06	1.39	345.03
7	269	1543896	3.66	1.48	366.94
8	242	706241.6	2.44	1.47	362.80
9	272	664606	2.51	1.56	386.22
10	300	922959.1	2.81	1.35	333.23
11	243	752198.5	3.21	1.43	352.94
12	281	727316.8	2.99	1.34	330.86
13	248	942287.7	3.11	1.46	360.31
14	285	1163233	3.10	1.36	336.67
15	244	1254023	3.28	1.39	344.02
16	281	966358.6	3.09	1.52	375.55
17	277	1180744	3.19	1.37	338.92
18	259	1798959	3.40	1.52	375.08
19	302	1506518	3.13	1.29	319.41
20	248	1478650	3.41	1.43	352.94
21	266	1332230	3.09	1.23	304.53
22	283	985658.9	3.06	1.33	329.58
23	270	1669877	3.33	1.47	364.66
24	289	1132115	2.82	1.50	372.38
25	232	1099777	3.32	1.34	332.03
26	302	1250070	3.17	1.41	348.46
27	278	1391273	3.19	1.44	357.35
28	271	1476892	3.00	1.29	320.00
29	242	1386178	3.33	1.27	314.82
30	293	1328424	3.30	1.40	346.48
31	242	1131126	3.28	1.35	333.15

Table S3
The results of spall experiments on peak-aged Z5 alloy including thickness (μm), strain rate (s^{-1}), shock stress (GPa), spall strength (GPa) and pullback velocity (m/s).

Shot No.	Thickness (μm)	Strain rate (s^{-1})	Shock stress (GPa)	Spall strength (Gpa)	Pullback (m/s)
1	203	2217417	3.67	1.58	389.85
2	203	1690616	3.27	1.45	359.02
3	198	2798721	3.35	1.81	447.58
4	207	2683489	3.42	2.21	546.11
5	206	1890421	3.19	1.37	338.15
6	206	3056042	3.88	1.99	493.58
7	193	2476961	3.42	1.29	319.65
8	213	830342.5	3.51	1.51	374.15
9	185	1034565	3.12	1.44	356.61
10	253	1182466	3.15	1.42	352.03
11	249	1313375	3.27	1.57	388.32
12	236	1028951	3.48	1.54	382.36
13	214	1440590	3.47	1.41	348.92
14	212	1133202	3.12	1.21	299.42
15	200	986129.5	2.56	1.36	335.89
16	203	979103.3	2.61	1.09	269.49
17	236	407457.7	1.73	0.83	204.18
18	197	618610.5	2.64	0.97	240.97
19	203	363273.1	1.39	0.48	117.63
20	199	1001282	2.53	1.22	301.16
21	258	1038576	3.29	1.49	368.96
22	207	862159.5	3.06	1.65	408.55
23	216	1411300	3.26	1.50	370.82
24	218	1347976	3.42	1.46	360.61
25	236	1387231	3.55	1.21	298.53
26	208	1200006	3.80	1.61	397.71

1.4. Additional Experimental Details

1.4.1. Additional TEM Micrographs

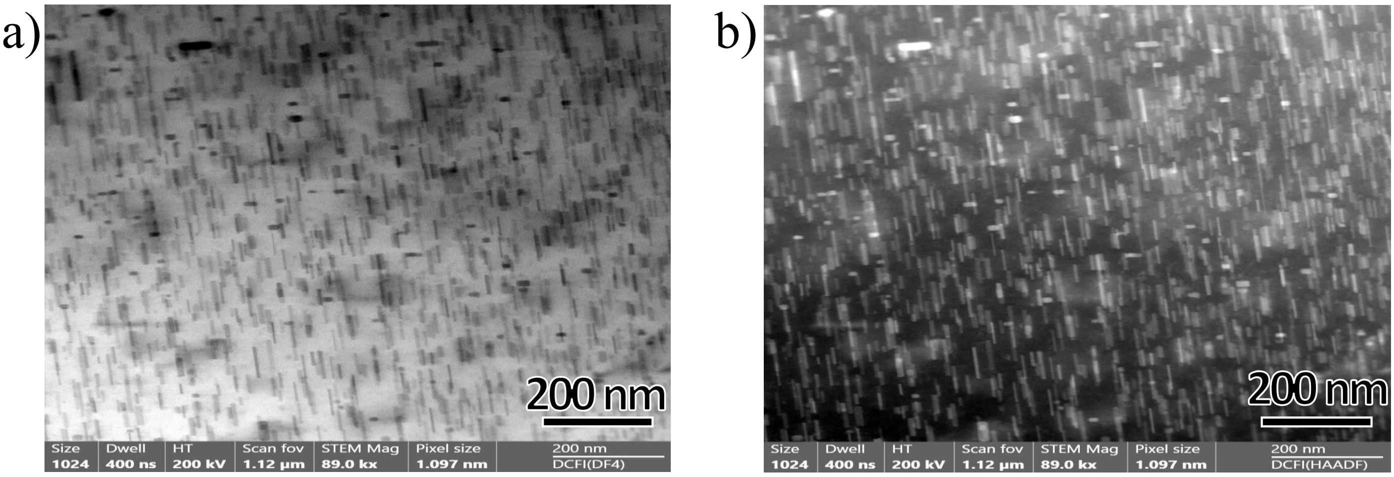

FS 7: STEM images of a) Z5 peak-aged observed under bright field microscope and b) Z5 peak-aged observed under dark field microscope.

1.4.2. Standard deviation from the Nanoindentation experiments

Table S4
Standard deviation of Quasistatic hardness for solutionized Z5 and peak-aged Z5 samples.

Solutionized Z5			Peak-Aged Z5		
S.No	Strain Rate (s^{-1})	Quasistatic Hardness_std	S.No	Strain Rate (s^{-1})	Quasistatic Hardness_std
1	0.01	0.044385	1	0.01	0.097712
2	0.1	0.063733	2	0.1	0.091532
3	1	0.065156	3	1	0.057564

Table S5
Standard deviation of Dynamic hardness for Solutionized Z5 sample.

S.No	Strain Rate (s^{-1})	Dynamic Hardness_STD	S.No	Strain Rate (s^{-1})	Dynamic Hardness_STD
1	10.5368	0.01615	61	1006.83789	0.09357
2	11.87996	0.08946	62	1058.67786	0.10125
3	13.71746	0.06307	63	1112.73409	0.10189
4	15.63303	0.0629	64	1168.54589	0.10428
5	16.9499	0.10939	65	1224.95179	0.10851
6	19.38332	0.09094	66	1285.555	0.11345
7	21.26067	0.06215	67	1345.54054	0.11029
8	23.5918	0.06152	68	1409.77242	0.11027
9	26.25462	0.06137	69	1479.21582	0.11207
10	29.31402	0.15121	70	1550.52019	0.11519
11	31.36326	0.07808	71	1621.00242	0.12046
12	35.12035	0.07173	72	1692.96066	0.11825
13	38.91717	0.09452	73	1769.79048	0.11893
14	42.93214	0.10059	74	1850.61845	0.11277
15	47.28611	0.11147	75	1935.71226	0.12544
16	51.71428	0.12663	76	2020.78274	0.12857
17	57.09975	0.09618	77	2109.09653	0.12327
18	61.56953	0.07985	78	2201.42923	0.12732
19	67.49483	0.08892	79	2298.89449	0.12484
20	73.44082	0.09063	80	2394.89766	0.11944
21	79.09578	0.09768	81	2495.77787	0.12956
22	86.77363	0.07735	82	2605.74169	0.13207
23	92.97928	0.08211	83	2711.33234	0.12492
24	102.04513	0.09434	84	2824.85956	0.12661
25	110.29707	0.11099	85	2941.44724	0.11752
26	117.91848	0.09071	86	3061.39759	0.11652
27	127.6346	0.09414	87	3182.59563	0.12828
28	137.35956	0.10321	88	3315.34195	0.11445
29	148.41189	0.09947	89	3443.04218	0.12567
30	158.94798	0.09437	90	3582.2955	0.12406
31	170.66804	0.08901	91	3718.89783	0.12712
32	184.3826	0.10115	92	3869.18493	0.13725
33	196.7869	0.08587	93	4017.25116	0.12485
34	210.43677	0.08737	94	4168.54847	0.12696
35	225.56907	0.09862	95	4331.43286	0.12761
36	240.88302	0.09293	96	4493.13283	0.13195
37	256.87634	0.07142	97	4660.26291	0.14771
38	275.35135	0.0887	98	4837.76617	0.13474
39	293.73678	0.08964	99	5017.62166	0.13879
40	311.97289	0.08487	100	5189.481	0.13698
41	331.23519	0.08369	101	5380.76496	0.14203
42	352.6805	0.09294	102	5583.12942	0.13622
43	376.23075	0.08993	103	5783.47385	0.14555
44	398.6735	0.09228	104	5975.85655	0.14192
45	423.94248	0.08565	105	6191.0202	0.13435
46	449.21152	0.08802	106	6417.74146	0.14723
47	475.82454	0.09796	107	6639.16311	0.16445
48	504.91543	0.08404	108	6874.66909	0.15783
49	533.0923	0.0842	109	7102.18887	0.16578
50	564.68611	0.08471	110	7347.30777	0.15691
51	596.11318	0.08891	111	7594.78445	0.13629
52	629.66989	0.08598	112	7854.36082	0.1576
53	665.20734	0.09349	113	8109.97729	0.16195
54	702.49227	0.0911	114	8382.98127	0.16341
55	740.72401	0.09057	115	8649.03082	0.16926
56	780.88126	0.09311	116	8945.83198	0.18487
57	822.83684	0.10031	117	9235.93133	0.17883
58	865.98754	0.10064	118	9540.39072	0.16098
59	911.66121	0.09852	119	9841.1845	0.19399
60	959.20555	0.09646			

Table S6
Standard deviation of Dynamic hardness for peak-aged Z5 sample.

S.No	Strain Rate (s^{-1})	Dynamic Hardness_STD	S.No	Strain Rate (s^{-1})	Dynamic Hardness_STD
1	11.4317	0	60	9847.84811	0.13541
2	13.6059	0.01506	61	959.09597	0.07525
3	15.34068	0.07662	62	1006.28321	0.07614
4	17.09732	0.08133	63	1057.7589	0.07772
5	18.93417	0.04807	64	1111.99088	0.07028
6	21.22634	0.02831	65	1169.34094	0.07438
7	24.0556	0.07375	66	1224.38848	0.07151
8	26.33735	0.0614	67	1284.33602	0.07956
9	29.12948	0.04923	68	1346.84544	0.07384
10	32.66838	0.06954	69	1413.14168	0.07135
11	34.83625	0.07593	70	1480.21105	0.07279
12	39.06283	0.07651	71	1547.48288	0.07005
13	42.68435	0.06671	72	1619.76735	0.06744
14	46.66146	0.06548	73	1697.59205	0.07034
15	51.6477	0.061	74	1771.03775	0.07
16	56.48525	0.07657	75	1850.10272	0.06852
17	61.35494	0.07169	76	1936.11215	0.06958
18	66.7823	0.06858	77	2021.17417	0.07083
19	72.75592	0.07499	78	2108.78121	0.07107
20	79.22108	0.07385	79	2199.38027	0.06533
21	86.62227	0.07211	80	2296.34924	0.07429
22	93.20207	0.06887	81	2398.49568	0.07144
23	101.08401	0.07093	82	2494.59703	0.06693
24	110.4806	0.08604	83	2603.17046	0.07155
25	118.78563	0.07363	84	2711.5216	0.0709
26	126.10944	0.08027	85	2822.9624	0.07366
27	137.75045	0.08467	86	2941.70274	0.0729
28	148.36915	0.06979	87	3058.78717	0.0659
29	159.36976	0.08922	88	3184.64014	0.07176
30	170.63119	0.08195	89	3312.5738	0.07171
31	184.15915	0.06833	90	3443.66464	0.06909
32	196.82152	0.06965	91	3578.73639	0.07519
33	210.05017	0.0644	92	3722.1544	0.07387
34	224.44571	0.07862	93	3864.67708	0.07607
35	241.10177	0.05486	94	4013.35726	0.07265
36	258.33593	0.07631	95	4174.19533	0.07723
37	274.58269	0.06617	96	4323.27745	0.07867
38	292.54554	0.07678	97	4490.96335	0.07297
39	312.2643	0.06738	98	4661.79432	0.07392
40	331.84418	0.0753	99	4826.88121	0.0823
41	352.08983	0.06947	100	5002.43394	0.08408
42	374.76696	0.07932	101	5190.53005	0.08859
43	398.12235	0.06503	102	5389.91886	0.08471
44	422.10922	0.06256	103	5577.82261	0.07853
45	448.38299	0.08049	104	5782.94352	0.0954
46	476.39404	0.07129	105	5984.84442	0.09328
47	503.35696	0.07023	106	6191.55544	0.09271
48	533.48216	0.07662	107	6410.68876	0.09653
49	565.34942	0.07533	108	6645.10721	0.09031
50	596.27527	0.07716	109	6868.25354	0.10244
51	630.23991	0.07234	110	7099.88638	0.09946
52	664.73547	0.07376	111	7352.04756	0.09845
53	703.19504	0.07361	112	7596.21588	0.09558
54	741.68297	0.07765	113	7850.56626	0.09473
55	779.67713	0.07314	114	8104.09223	0.1263
56	822.55709	0.08055	115	8385.46575	0.09898
57	868.25771	0.07562	116	8665.08226	0.10278
58	910.25145	0.06809	117	8942.38358	0.10007
59	9530.94562	0.11103	118	9245.37519	0.10506

Terminology

θ	Angle	τ_s	Resolved shear stress
A	Area	G_m	Shear modulus
C_0	Bulk wave-speed	γ	Shear strain
b	Burger's vector	τ	Shear stress
$\dot{H}$	Change in hardness over time	Σ^*	Spall strength
τ_r	Critical resolved shear stress	ϵ	Strain
N_1	Density of grain boundary	n	Strain hardening exponent
N_2	Density of particle nucleation sites	$\dot{\epsilon}$	Strain rate
N	Density of Potential Nucleation sites	B	Strain rate hardening modulus
r_0	Dislocation core radius	τ_{ss}	Strengthening via distortion of lattice
ρ	Dislocation density	M_S	Taylor factors for random solutionized Z5
δ	Elastic-Plastic correction factor	M_{P-A}	Taylor factors for weakly textured peak-aged Z5
G	Formation energy	T	Temperature
d_s	Glide plane spacing of precipitates	β_0	Thermal-softening coefficient
l	Grain size	U_{fs}	Velocity drop
k	Hall-petch slope	V	Volume
H	Hardness	σ_Y	Yield sterngth
h	Indentation depth	E	Young's modulus
$\dot{h}$	Indentation depth over time	**Alloy Designations (All at.%)**	
$\dot{\epsilon}_i$	Indentation strain rate		
w	Interaction energy	Zn	n% Zn, rest Mg
λ_e	Inter-particle spacing	**Abbreviations**	
P	Load		
$\dot{P}$	Loading rate	ADF	Annular dark-field
c	Nominal concentration	CSM	Continous Stiffness Measurement
I	Nucleation rate	CRSS	Critical Resolved Shear Stress
r_p	Planar radius	DOE	Diffractive Optical Element
ν	Poisson's ratio	EFL	Effective Focal Length
d_t	Precipitate diameter ($= 2r_p$)	EBSD	Electron Backscatter Diffraction
t_t	Precipitate thickness	GP zone	Guinier–Preston zone
f	Precipitate volume Fraction	HCP	Hexagonal close-packed
$\mathcal{R}_y$	Probability distribution function	LDMF	Laser Driven Micro-Flyer
σ_G	quasistatic strength	LIPIT	Laser Induced Particle Impact Test
r	Radial distance	PDV	Photonic Doppler Velocimetry
R	Radius	RMS	Root Mean Square
β_1	Rate-sensitivity parameter	SEM	Scanning Electron Microscope
ρ_0	Reference density	STEM	Scanning Transmission Electron Microscopy
l_0	Reference grain size	TEM	Transmission Electron Microscopy
d_s^0	Reference mean particle spacing	XRD	X-ray Diffraction
$\dot{\epsilon}_0$	Reference strain rate		

References

[1] Johnson, G. R. A constitutive model and data for metals subjected to large strains, high strain rates and high temperatures. In *Proceedings of the 7th International Symposium on Ballistics, The Hague, Netherlands, 1983* (1983).

[2] Huh, H. & Kang, W. Crash-worthiness assessment of thin-walled structures with the high-strength steel sheet. *International journal of vehicle design* **30**, 1–21 (2002).

[3] Cowper, G. R., Symonds, P. S. *et al.* Strain-hardening and strain-rate effects in the impact loading of cantilever beams (1957).
